# Evaluating functional brain organization in individuals and identifying contributions to network overlap

**DOI:** 10.1162/imag_a_00046

**Published:** 2023-12-08

**Authors:** Janine D. Bijsterbosch, Seyedeh-Rezvan Farahibozorg, Matthew F. Glasser, David Van Essen, Lawrence H. Snyder, Mark W. Woolrich, Stephen M. Smith

**Affiliations:** a Department of Radiology, Washington University School of Medicine, Saint Louis, Missouri, United States.; b Wellcome Centre for Integrative Neuroimaging (WIN FMRIB), Oxford University, Oxford, United Kingdom; c Department of Neuroscience, Washington University School of Medicine, Saint Louis, Missouri, United States.; d Oxford Centre for Human Brain Activity, Wellcome Centre for Integrative Neuroimaging, Department of Psychiatry, University of Oxford, Oxford, United Kingdom

**Keywords:** resting-state functional MRI, brain networks, spatial overlap, individualized organization

## Abstract

Individual differences in the spatial organization of resting-state networks have received increased attention in recent years. Measures of individual-specific spatial organization of brain networks and overlapping network organization have been linked to important behavioral and clinical traits and are therefore potential biomarker targets for personalized psychiatry approaches. To better understand individual-specific spatial brain organization, this paper addressed three key goals. First, we determined whether it is possible to reliably estimate weighted (non-binarized) resting-state network maps using data from only a single individual, while also maintaining maximum spatial correspondence across individuals. Second, we determined the degree of spatial overlap between distinct networks, using test-retest and twin data. Third, we systematically tested multiple hypotheses (spatial mixing, temporal switching, and coupling) as candidate explanations for why networks overlap spatially. To estimate weighted network organization, we adopt the Probabilistic Functional Modes (PROFUMO) algorithm, which implements a Bayesian framework with hemodynamic and connectivity priors to supplement optimization for spatial sparsity/independence. Our findings showed that replicable individual-specific estimates of weighted resting-state networks can be derived using high-quality fMRI data within individual subjects. Network organization estimates using only data from each individual subject closely resembled group-informed network estimates (which was not explicitly modeled in our individual-specific analyses), suggesting that cross-subject correspondence was largely maintained. Furthermore, our results confirmed the presence of spatial overlap in network organization, which was replicable across sessions within individuals and in monozygotic twin pairs. Intriguingly, our findings provide evidence that overlap between 2-network pairs is indicative of coupling. These results suggest that regions of network overlap concurrently process information from both contributing networks, potentially pointing to the role of overlapping network organization in the integration of information across multiple brain systems.

## 1. INTRODUCTION

Recent studies have revealed substantial inter-individual variability in the spatial organization of the brain as measured with resting state functional MRI (rfMRI) (Braga & Buckner, 2017; D. Wang et al., 2015; Glasser et al., 2016; Gordon, Laumann, Adeyemo, & Petersen, 2017; Gordon, Laumann, Adeyemo, Gilmore, et al., 2017; Gordon, Laumann, Gilmore, et al., 2017; Laumann et al., 2015). Importantly, such inter-individual spatial variability in functional brain organization is strongly associated with behavioral traits (Bijsterbosch et al., 2018; Kong et al., 2019). The overarching objective of this paper is to characterize weighted (i.e., non-binarized) spatial organization of resting-state networks within individuals, with a specific focus on gaining insights into spatially overlapping network organization. We focus on network overlap because recent work has highlighted that individual differences in network overlap are strongly associated with behavior (Bijsterbosch et al., 2019). Yet, the complex spatially overlapping nature of network organization is largely underestimated and understudied in traditional rfMRI analysis approaches that enforce binary area/network assignment or encourage spatial independence of networks.

Identifying network organization at the individual level rather than using group information raises multiple challenges. First, individual estimates of network organization are noisier than group estimates, partly because group estimates benefit from collating large amounts of data. This challenge can be partially addressed by obtaining large amounts of data from each individual (precision functional mapping approach (Gratton et al., 2020)), but such extensive data acquisition may not be feasible in all participants and settings. Second, although a purely individual-specific set of network maps represents the most accurate and unbiased estimate of the individual’s brain organization (Gordon, Laumann, Gilmore, et al., 2017), it may lack network correspondence across individuals. Assuming the presence of cross-participant commonalities in their network structure, such group correspondence is valuable for network labeling and interpretability, and essential for group-level and between-subject analytical comparisons. Group-based estimates applied to individuals have built-in correspondence, but these individual estimates may be biased towards the group estimate (Bijsterbosch et al., 2019). Probabilistic Functional Modes (PROFUMO) is a Hierarchical Bayesian algorithm developed to try to optimize this trade-off by using group-level priors to achieve correspondence, whilst optimizing individual-specific estimates to maximize the accuracy of individual network maps (Harrison et al., 2015). Compared to other hierarchical network approaches such as Kong et al. (2019), PROFUMO estimates the group prior from the data (instead of adopting an atlas) and includes a group prior on the temporal connectivity matrix (for a comprehensive overview of hierarchical model differences, please see Appendix 1 for Farahibozorg et al., 2021). Although PROFUMO has been successfully applied in group data (Bijsterbosch et al., 2018; Farahibozorg et al., 2021; Harrison et al., 2020), an open question is whether it can be robustly applied to data from only a single individual without sacrificing correspondence, which is of particular interest in the context of personalized psychiatry and translational work in non-human primates and other animal models. The first goal of this paper was to determine whether PROFUMO can reliably estimate weighted (i.e., non-binarized) resting-state networks using only data from a single subject, while also achieving substantial “non-enforced” correspondence across individuals (i.e., without data from other participants contributing to a group prior to inform correspondence).

Spatial overlap between rfMRI networks beyond classical “hub regions” has been observed across a variety of analytical brain representations (Lee et al., 2016; Lin et al., 2018; Najafi et al., 2016), and has been linked to behavioral traits (Bijsterbosch et al., 2019). PROFUMO accurately estimates spatial overlap in rfMRI network organization (Bijsterbosch et al., 2019), which is a key advantage compared to approaches that aim for a “hard” binarized parcellation or approaches that enforce spatial independence between networks (Bijsterbosch et al., 2020). Despite broad interest in “hub” regions within the graph theory domain that typically adopts hard parcellations (Bertolero et al., 2018; Buckner et al., 2009; Warren et al., 2014), a detailed spatial investigation into individual-specific overlap between weighted estimates of network organization is lacking. Studying the overlapping properties of individualized brain networks is of interest because network overlap may be a sensitive marker for use in personalized psychiatry settings (Gratton et al., 2020; Insel, 2014; Williams, 2016) given prior evidence of behavioral relevance (Bijsterbosch et al., 2019), provided that it can be robustly and reliably detected in individuals. Furthermore, this work contributes to the broader literature on precision functional mapping (Gordon, Laumann, Gilmore, et al., 2017; Gratton et al., 2018; Poldrack, 2017; Poldrack et al., 2015). The second goal of this paper was to characterize network overlap among weighted individual-specific resting state networks estimated using PROFUMO.

**Fig. 1.**
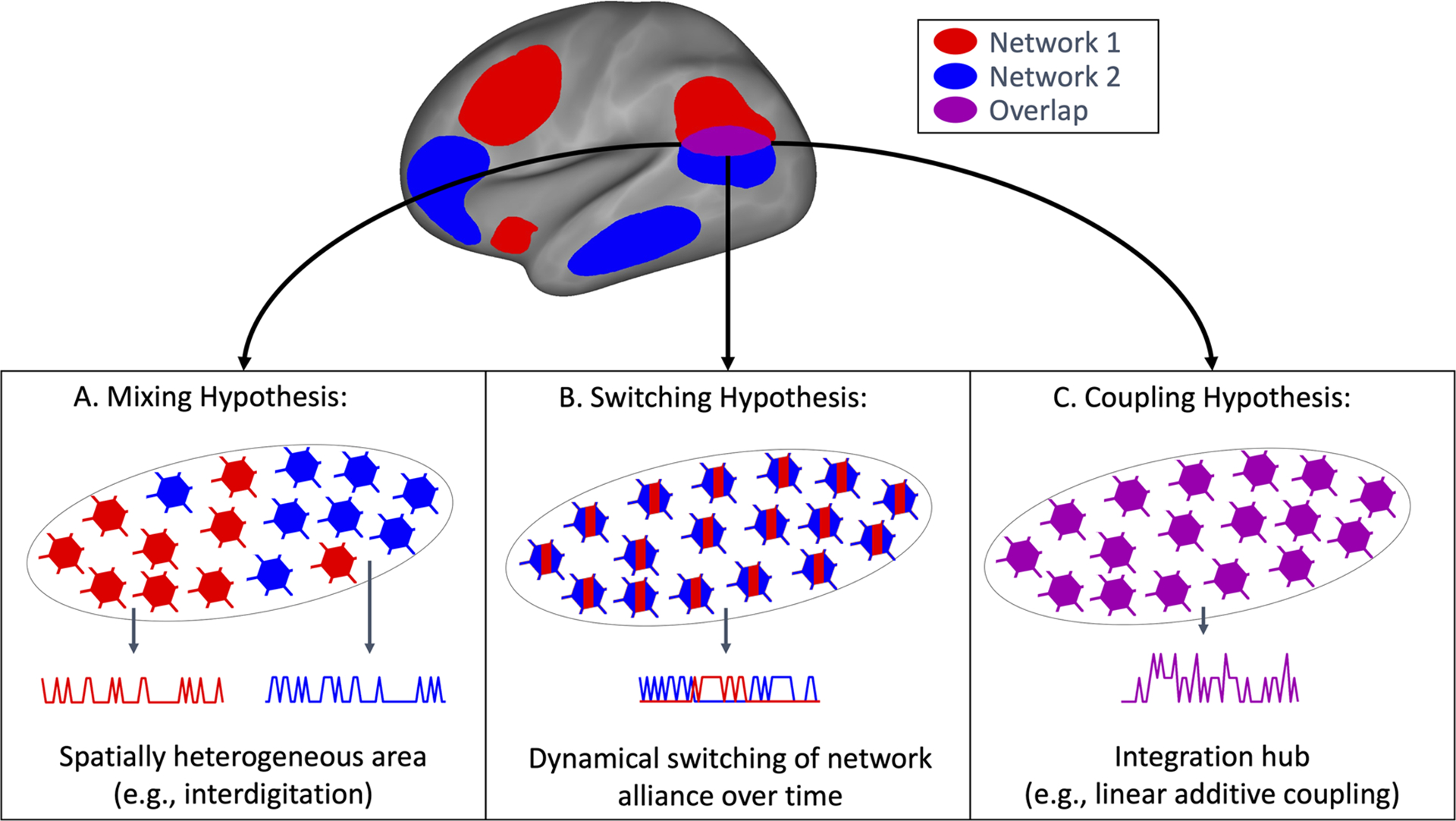
Graphical summary of the three hypotheses of network overlap. (A) The spatial mixing hypothesis suggests that individual vertices (illustrated as neurons) within a region of network overlap may be part of either Network 1 (red) or Network 2 (blue). (B) The dynamic switching hypothesis suggests that a region of network overlap may be spatially homogeneous but may switch network allegiance between Network 1 (red) and Network 2 (blue) over time. (C) The coupling hypothesis suggests that a region of network overlap may integrate information from both Networks 1 and 2.

Each type of resting-state fMRI analysis (e.g., using different approaches such as Independent Component Analysis, graph theory, amplitude of low frequency fluctuations, PROFUMO, etc.) provides a different low-dimensional representation of the dataset (Bijsterbosch et al., 2020). Although optimized to best fit the data (Bijsterbosch et al., 2021), these brain representations (i.e., summary measures derived from different approaches to rfMRI data, such as connectivity matrices, network maps, amplitude maps, etc.) are necessarily lossy given the intrinsic goal of dimensionality reduction. In the case of PROFUMO, a set of stationary large-scale modes of brain organization are derived that collapse fine-grained spatial structure and simplify temporally dynamic processes. As such, there are multiple potential mechanisms that may give rise to spatially overlapping network organization as observed between network maps estimated with PROFUMO. First, it is possible that a brain region in which two networks appear to overlap may, in fact, be a spatially heterogeneous mixture of cortical patches that are individually part of either network 1 or network 2, implying no real functional “link” between the two networks as a result of the overlap (Fig. 1A). Such a spatially heterogeneous overlap region may, for example, result from network interdigitation (Braga & Buckner, 2017; Braga et al., 2019), or regional gradients (Blazquez Freches et al., 2020; Haak et al., 2018). Second, the region of network overlap may dynamically switch its network allegiance over time to be part of either network 1 or network 2 at any given moment (Hutchison et al., 2013) (Fig. 1B). Such dynamic switching would appear as a spatially overlapping network structure given the stationary (time-averaging) nature of the PROFUMO model. Third, network overlap might indicate that signals from network 1 and network 2 are jointly processed and “deeply functionally integrated” within regions of network overlap (Fig. 1C). This third hypothesis is perhaps the most intriguing as it may indicate information coupling involving a specific functional role of overlap regions, and contributing to between-network communication. The third goal of this paper was to systematically compare these spatial mixture, dynamic switching, and coupling hypotheses of network overlap.

In this paper, we leveraged a unique subset of the Human Connectome Project Young Adult data (Van Essen et al., 2013), by focusing on individuals who underwent three complete resting-state sessions (3 T, 3 T re-scan, and 7 T) for a total of approximately 3 hours of rfMRI data per individual. The resulting sample of N = 20 that met this criteria further included 8 monozygotic twin pairs, providing a rich cohort to investigate individual-specific weighted network organization. Our focus on a small sample of densely sampled individuals was informed by the interest in individual-specific network organization. Although no brain-behavior associations were feasible given the small sample (Marek et al., 2022), prior work has extensively studied individual differences in PROFUMO, including behavioral associations with spatial organization (Bijsterbosch et al., 2018), heritability (Harrison et al., 2020), and network variants as a function of dimensionality (Farahibozorg et al., 2021). The results of this work support the application of PROFUMO weighted networks in individual participants, which paves the way for future applications in a personalized psychiatry framework. Furthermore, our findings suggest a coupling mechanism underlying spatially overlapping network organization, which supports the hypothesis that regions of network overlap play an important functional role in terms of cross-network coupling (Gordon et al., 2018).

## 2. METHODS

### 2.1. Dataset

We used high-quality data from the Human Connectome Project (HCP) (Van Essen et al., 2013), focusing on N = 20 individuals (including 8 monozygotic twin pairs) who underwent a complete set of four 3 T, four 7 T, and four retest 3 T runs, thereby accumulating approximately 3 hours (13,200 timepoints) of rfMRI data across 12 scans per individual. This HCP-YA sub-sample was 80% female with a mean age of 30.1 years (standard deviation = 3.84; range 22–34). Briefly, the 3 T rfMRI data were acquired at 2 mm isotropic voxel size using a multiband factor of 8, a TR of 0.72 seconds, and a TE of 33 ms; the 7 T rfMRI were acquired at 1.6 mm isotropic voxel size, a multiband acceleration of 5, in-plane acceleration 2, a TR of 1.0 seconds, and a TE of 22.2 ms (see further details in Smith et al., 2013; Van Essen et al., 2013; Vu et al., 2017). Data were preprocessed using the HCP minimal processing pipelines (Glasser et al., 2013). All 3 T and 7 T data were analyzed in the Connectivity Informatics Technology Initiative (CIFTI) format, which consists of 91,282 grayordinates with approximately 2-mm spatial resolution (i.e., 7 T data were downsampled to match the 3 T spatial resolution). ICA-FIX was then applied to remove structured noise (Griffanti et al., 2014; Salimi-Khorshidi et al., 2014), and data were aligned using multimodal surface matching (MSM-All; Robinson et al., 2014) to align areal features (myelin and RSNs).

### 2.2. PROFUMO estimation

PROFUMO is a matrix factorization approach for the estimation of resting-state networks that adopts spatial priors, temporal priors, and a noise model in a hierarchical Bayesian model (see Harrison et al., 2015, 2020 and Supplementary Tables S1 and S2). PROFUMO spatial maps are calculated by vertex-wise multiplying the probability (0–1; the probability that a given weight is drawn from the signal rather than the noise distribution) by the estimated mean (derived from Gaussian mixture model). PROFUMO was applied in several distinct ways:

1. Classic group-PROFUMO was performed in which data from all 12 runs across all 20 participants were used and modeled hierarchically according to the levels of subjects and (beneath that) runs. Notably, this version of PROFUMO is recommended for studies that include only one or a small number of sessions per individual.
2. Single-subject PROFUMO was performed independently for each of the 20 participants, using all 12 runs for each, those 12 being considered as separate “subjects” in the estimation of PROFUMO’s Gaussian mixture model (Harrison et al., 2020). As such, the “group” priors for single-subject PROFUMO here (and in #4 & #5 below) reflect the single-subject averages, such that no data/information from other participants was used in these analyses.
3. Classic group-PROFUMO was performed similar to case (#1), using 12 individual runs from 12 separate participants. This was in order to obtain a group-level estimation of modes using the same amount of data available for the separate subject runs (case (#2), i.e., matching the effective signal to noise ratio; SNR).
4. Test-retest single-subject PROFUMO was performed independently for each of the 20 participants and independently using two sets of 6 runs each (split evenly across 3 T, 7 T, and retest data; and always including a pair of opposite phase-encode directions).
5. Single-subject PROFUMO similar to case (#2) was performed stepwise on cut-down versions of the 12 runs that included a progressively increasing number of timepoints (in increments of 1/12th of the run timepoints) to determine how much data are needed to obtain reliable network estimates. This approach was taken to ensure that all PROFUMO runs included equal contributions from 3 T, retest, and 7 T data across all phase-encode directions.

All of the PROFUMO runs were performed at a dimensionality of 20 to focus on the spatial organization of large-scale resting-state networks. Furthermore, classic group-PROFUMO (#1) was repeated at dimensionalities 15, 30, 40, and 50 to determine the stability of the selected networks across PROFUMO decomposition dimensionalities. Beyond the key parameter of dimensionality (number of networks), all PROFUMO parameters were set to the default. A summary of all PROFUMO parameters, hyperparameters, and hyperpriors can be seen in Supplementary Tables S1 and S2. Beyond these PROFUMO parameters, additional key measures of interest for this paper include the similarity between group and individual network estimates indicative of correspondence and the test-retest reliability of network spatial maps representative of stable and reliable network estimates (described further below in Sections 2.3 and 2.4).

### 2.3. PROFUMO mode selection

The Hungarian algorithm (a.k.a. “munkres” algorithm) was used to reorder PROFUMO modes (i.e., “networks”) for each of the single-subject runs (#2 above), the split 1 and split 2 single-subject runs (#4 above), and the 12-run group run (#3 above) to best-match the mode order obtained from the full group results. Briefly, the Hungarian algorithm solves the assignment problem by permuting rows of the network-to-network spatial correlation matrix to minimize the trace of the permuted cost matrix (Kuhn, 1955; Munkres, 1957). For each of the 20 modes, we estimated the test-retest correlation per subject as the Pearson correlation across all 91,282 CIFTI grayordinates between split 1 and split 2 single-subject runs. For each of the 20 modes, we also estimated the subject-group correlation per subject as the Pearson correlation across all 91,282 vertices between the map from the subject run (#2 above) and the subject-specific estimated map from the group run (#1 above). This “group-individual run” measure addresses the question of correspondence across individuals because it compares classic group PROFUMO modes (which benefit from explicit correspondence through the group prior in the hierarchical Bayesian algorithm) to single-subject PROFUMO modes (which do not involve any group information). Modes that achieved both a median (across subjects) test-retest correlation of 0.7 or greater and a median (across subjects) group-individual correlation of 0.7 or greater were used in subsequent analyses. Notably, 0.7 was previously recommended as a default lowest acceptable standard of reliability in basic research (Nunnally, 1967). Out of the 20 PROFUMO modes, 12 modes met these requirements. Within these 12 modes, individual participants’ missing modes were defined as modes with both a test-retest correlation lower than 0.2 and a subject-group correlation lower than 0.2 (both estimated at the subject level). Missing modes were ignored in subsequent analyses. For naming purposes, modes were spatially mapped onto the Yeo-7 parcellation (Yeo et al., 2011) and we followed the network naming taxonomy suggested by Uddin et al. (2019).

### 2.4. PROFUMO mode stability

We assessed the stability of spatial maps in several ways. As described in Section 2.3, the first two measures of mode stability were within-participant *test-retest reliability* between splits 1 and 2, and *within-subject similarity (group-individual run)* correlations between the subject run (#2 above) and the subject estimates obtained in the group run (#1 above). We also tested *twin similarity (individual runs)* and *twin similarity (group run)* by correlating each PROFUMO mode within each monozygotic twin pair using modes from separate subject runs (#2 above) or subject-specific estimates from the group run (#1 above), respectively. For comparison, we also investigated *between-subject similarity (individual runs)* and *between-subject similarity (group run),* reflecting the same across-subject correlations for all possible non-twin pairs of participants. All mode stability measures were performed on each spatial map (correlation across 91,282 vertices), on the temporal connectivity matrix (correlation across the 66 edges in the lower triangle, where edges represent the partial correlation between mode timeseries), and on the spatial overlap matrix (correlation across the 66 edges in the lower triangle, where edges represent the correlation between mode maps; see Section 2.5 and Bijsterbosch et al., 2019).

### 2.5. Spatial overlap measures

To quantify spatial overlap across all 12 modes, we generated a spatial overlap matrix by estimating the Pearson’s correlation coefficients across grayordinates between all possible pairs across the 12 network maps, as developed in Bijsterbosch et al. (2019). Correlation coefficients were z-transformed prior to averaging across individuals. Stability of individual specific spatial overlap matrices was calculated as described in Section 2.4. Importantly, this spatial overlap matrix definition of network overlap offers a threshold-free estimation of overlapping network organization.

To estimate spatial maps of network overlap in individual participants, each mode was binarized using a threshold of 1, and the number of overlapping modes were counted at each vertex. Each vertex was multiplied by its corresponding cortical area to estimate the spatial overlap area shown in Figure 7B. A threshold of 1 represents the 96.5th percentile across all map weights and therefore offers a relatively conservative estimate of spatial overlap that is driven only by vertices with strong network contributions.

### 2.6. Focusing on 2-network overlap

**Fig. 2.**
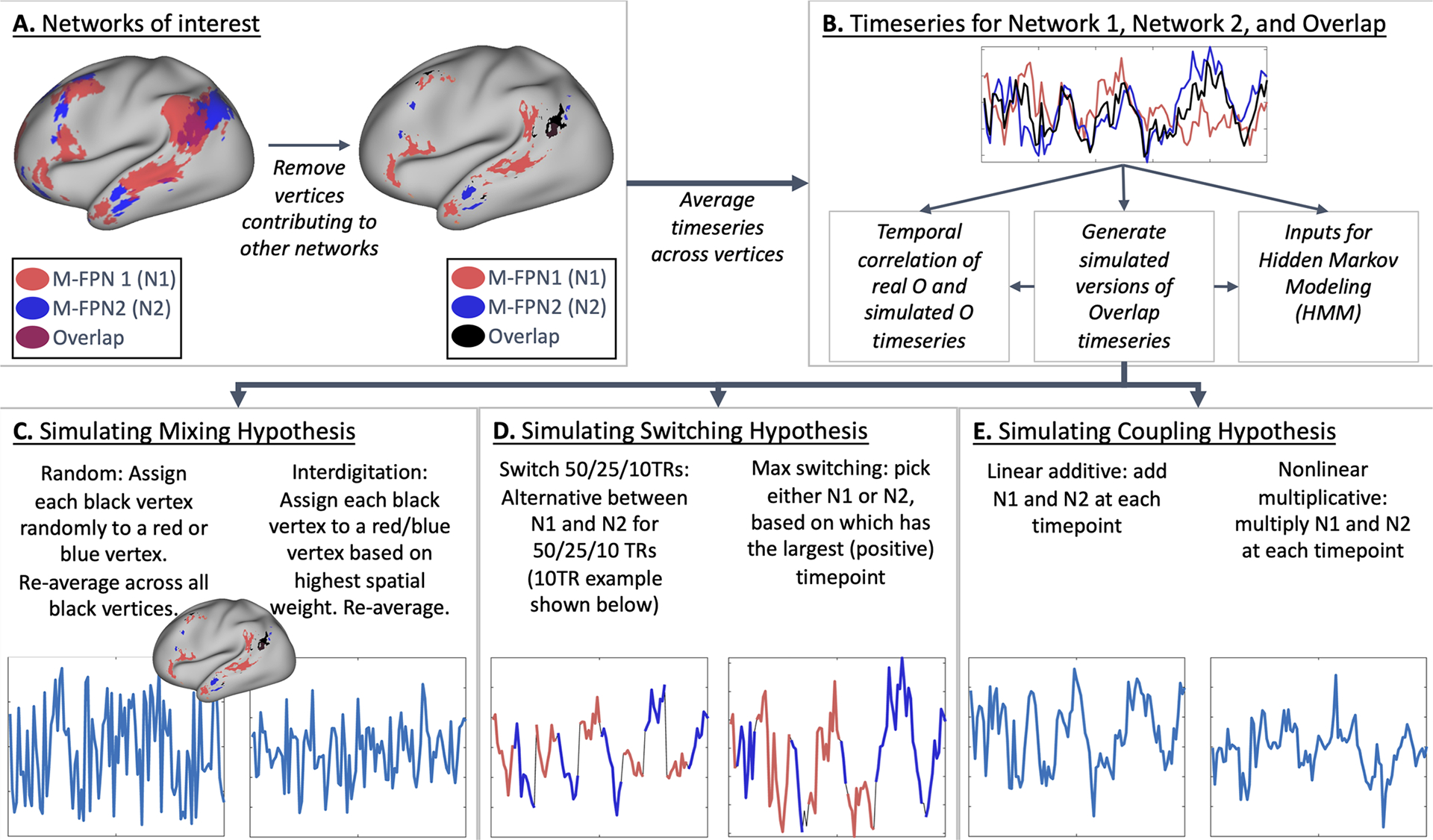
Graphical summary of overlap hypothesis testing methodology. (A) Two spatially overlapping networks are selected and vertices are assigned to either network 1, network 2, or network Overlap. Vertices contributing to additional networks are excluded. (B) Average timeseries are extracted per subject, per run for Network 1, Network 2, and Overlap. (C) Mixing hypothesis-based semi-simulated (i.e., using actual timeseries from non-overlap vertices) versions of the Overlap timeseries are generated based on random and interdigitation-based mixtures of Network 1 and Network 2 vertices. (D) Switching hypothesis-based semi-simulated versions of the Overlap timeseries are generated based on concatenated segments from Network 1 and 2 timeseries. (E) Coupling hypothesis-based semi-simulated versions of the Overlap timeseries are generated based on additive or multiplicative combinations of Network 1 and 2 timeseries.

To systematically compare our hypotheses regarding network overlap, we focused on spatial overlap between pairs of networks. For each individual and each possible network pair (12*11/2 = 66), we identified vertices uniquely associated with network 1 as those vertices with a weight of 1 or greater for network 1, a weight of less than 0.1 for network 2, and a summed weight across all other 10 modes of less than 0.1 (Fig. 2B). Negative map weights were not included in the estimation of network overlap, because the inclusion of negative vertices would dilute averaged timeseries by canceling out positive vertices. A similar procedure was used to identify vertices uniquely associated with network 2. To locate the spatial overlap region, we identified vertices having a weight of 1 or greater for both network 1 and network 2 and a summed weight across all other 10 modes of less than 0.1. To ensure that findings were not driven by specific thresholding, all nine possible pairings between three thresholds for network vertex weights [0.75 1 1.25] and three thresholds for summed weights [0.02 0.1 0.5] were tested. This procedure was performed using mode maps estimated from the individual subject PROFUMO runs (#2 above). PROFUMO spatial map values in our data ranged from −3.6 to 7.1, and the applied threshold of 1 represents the 96.5th percentile. Hence, thresholds were applied to the PROFUMO spatial maps as estimated by the PROFUMO algorithm without further transforms. As such, we focused specifically on two-mode overlap vertices, by removing vertices with significant contributions of additional modes (Fig. 2A). We focus on 2-network overlap because it offers a tightly controlled test-bed for the hypothesis testing element of our work. Future work may expand into more complex overlapping network organization. Overlap regions were defined based on the data-driven approach described above without any explicit exclusions, and the resulting overlap patterns (see Supplementary Fig. S1) comprehensively cover all areas of potential interest.

The number of network pairs for further investigation was reduced from 66 (all possible pairs) by selecting only those networks pairs in which the spatial overlap regions contained at least 25 vertices for at least half of the participants (n >= 10). This network pair selection was performed to focus on pairs with robust and replicable overlap, and to reduce the computational demands for subsequent analyses. Out of the 66 possible network pairs, 20 pairs were selected for further analysis (see Supplementary Fig. S1).

For each of the 20 network pairs, three summary timeseries were calculated (Fig. 2B) by averaging across: all vertices uniquely associated with network 1 (“N1”), all vertices uniquely associated with network 2 (“N2”), and all vertices in the overlap region (“O”). Each timeseries was standardized to a mean of zero and a standard deviation of 1 within each of the 12 runs. Any participants who did not have any vertices in the overlap region based on these criteria were excluded from subsequent analyses.

### 2.7. Generating semi-simulated data for the overlap region

Multiple mechanistic hypotheses might in principle explain the occurrence of spatial overlap in stationary maps estimated using PROFUMO, and it is currently unknown what drives the observed (apparent) spatial overlap. To address this issue, we generated semi-simulated versions of overlap timeseries to test different hypotheses as described below. The semi-simulated version of overlap timeseries were compared to the original overlap timeseries using direct timeseries correlation, frequency characteristics (see Section 2.8 for further details), and by fitting a general linear model (GLM; see Section 2.9 for further details).

#### 2.7.1. Network switching hypothesis

One hypothesis is that the overlap region may dynamically switch network alliance between networks 1 and 2 over time within a scanning run (Fig. 1B). Such dynamic switching would appear as overlap when using stationary methods such as PROFUMO, which effectively average across time. To test this hypothesis, we semi-simulated four versions of the overlap timeseries using the real N1 and N2 timeseries for each participant and run:

- Switch 50 TRs (Fig. 2D) starts with the first 50 time-points from N1, then contains the second 50 time-points from N2, then the third 50 timepoints from N1 and so on. Notably, potential phase shifts between original and semi-simulated data (i.e., mismatches in the order between N1 and N2) may impact the correlation between the original and semi-simulated versions of the timeseries. However, such phase discrepancies would not impact our second comparison measure based on the GLM, because the resulting GLM beta weights will capture order effects.
- Switch 25 TRs (Fig. 2D) is the same as above, but switching between N1 and N2 every 25 timepoints.
- Switch 10 TRs (Fig. 2D) is the same as above, but switching between N1 and N2 every 10 timepoints.
- Max switching (Fig. 2D) assigns each timepoint in the semi-simulated overlap timeseries as the maximum from either N1 or N2 based on whichever datapoint (in real data timeseries N1 and N2) is higher for a given TR.

#### 2.7.2. Coupling hypothesis

Another hypothesis is that the overlap region is integrating data from both network 1 and network 2 at each TR (Fig. 1C). To test the coupling hypothesis, we semi-simulated two versions of the overlap timeseries using the real N1 and N2 timeseries for each participant and run:

- Linear additive coupling (Fig. 2E) is the sum of N1 and N2 within each TR.
- Nonlinear multiplicative coupling (Fig. 2E) takes the element-wise product between N1 and N2 after setting N1 and N2 to a minimum of zero (to avoid negative values) respectively.

#### 2.7.3. Spatial mixture hypothesis

A final hypothesis is that the overlap region is a spatial mixture of vertices linked to network 1 and network 2 (Fig. 1A), akin to the concept of network interdigitation (Braga & Buckner, 2017) or within-region gradients (Haak et al., 2018). To test this hypothesis, we semi-simulated two versions of the overlap timeseries using the real vertex timeseries uniquely associated with either network 1 or network 2. In contrast to the other semi-simulated versions of the overlap timeseries described above, this method does not use the mean N1 and N2 timeseries and instead repeats the averaging across vertices.

- Spatial random mixture (Fig. 2C) assigns half of the vertices in the overlap region to a randomly chosen vertex out of those uniquely associated with network 1 (with replacement) and assigns the other half of the vertices in the overlap region to a randomly chosen vertex out of those uniquely associated with network 2 (with replacement). The semi-simulated overlap timeseries is then averaged across all vertices and standardized as above.
- Spatial interdigitation (Fig. 2C) assigns each vertex in the overlap region based on whether the spatial weight for that vertex was higher for network 1 or for network 2. If the spatial weight for M-FPN 1 is higher, the timeseries of the vertex uniquely associated with network 1 with the grayordinate that is closest in vectorized indexing is assigned to that overlap vertex (with replacement). The semi-simulated overlap timeseries is then averaged across all overlap vertices and standardized as above. We refer to this option as “spatial interdigitation” because the PROFUMO spatial weights reflect spatially contiguous areas (see Supplementary Fig. S2).

Although the 7 T data were acquired at higher resolution than the 3 T data, we analyzed data on the same 32k mesh (i.e., matched resolution). A deeper investigation into high-resolution 7 T network organization is beyond the scope of this work.

### 2.8. Frequency characteristics of semi-simulated timeseries

Fourier transforms were performed on the original overlap timeseries and each of the eight semi-simulated overlap timeseries to compare the resulting frequency characteristics. Fourier transforms were performed separately for each participant and each run using only the 3 T runs (8 per individual) for ease of comparison due to matched TR and timeseries length. Resulting power spectra were normalized to a maximum of 1 for ease of comparison.

### 2.9. Hypothesis testing using the general linear model

The advantage of the semi-simulated timeseries approach above is that it offers a hypothesis-testing framework with direct comparisons across hypotheses based on timeseries correlations between the semi-simulated and original overlap timeseries. The general linear model (GLM) offers a complementary approach based on model fit. Advantages of the GLM approach include data-driven estimation of coefficients. These data-driven parameter estimates enable non-equal contributions from network 1 and network 2 in the linear coupling hypothesis, and can address ordering effects and window-duration challenges in the switching hypotheses. As such, we adopted a GLM framework to corroborate or refute results from the semi-simulated approach. For all GLMs below, the dependent and independent variables were all demeaned and variance normalized prior to model fit. The GLMs were fit separately for each participant and each network pair, and GLMs were also fit separately to data from each of the 12 runs. To enable model comparisons across hypotheses, we adopt adjusted *R*^2^ across a range of models described below. Importantly, the adjusted *R*^2^ is used to account for differences in model complexity.

#### 2.9.1. Network switching hypothesis

To test the switching hypothesis, two separate GLMs were fit to each windowed segment, using the following equations:

$$\text{Windows}\;\text{with}\;\text{Network}\;1\;\text{alliance:}\;O_{w}=\beta_{1}*N1_{w}$$

$$\text{Windows}\;\text{with}\;\text{Network}\;2\;\text{alliance:}\;O_{w}=\beta_{2}*N2_{w}$$

Here, *O* refers to the overlap timeseries, *N*1 refers to the timeseries from network 1, and *N*2 refers to the timeseries from network 2 (all derived from the original data, averaged across vertices). *w* indicates the window, which is modeled as a sliding window of 10 timepoints shifted in steps of 1 timepoint. Note that there is no need to model longer sliding window durations, because any switching behavior in longer windows will be captured across multiple shorter windows. For each window, the adjusted *R*^2^ of the best performing model (either network 1 alliance or network 2 alliance) was recorded, and the resulting vector of *R*^2^ was averaged across windows. If the overlap region is dynamically switching alliance over time, then any individual segment should be well explained by either N1 or N2, and the resulting *R*^2^ (selected as the highest between the two models above) should therefore be equal to or higher to *R*^2^ for other GLMs described below.

#### 2.9.2. Coupling hypothesis

To test the coupling hypotheses, separate GLMs were fit to compare the linear, combined (including linear and nonlinear terms), and nonlinear coupling hypotheses. Models were fit to the overall timeseries using the equations:

$$\text{Linear}\;\text{hypothesis:}\;O=\beta_{1}*N1+\beta_{2}*N2$$

$$\text{Combined}\;\text{linear}\;\text{and}\;\text{nonlinear}\;\text{model:}\;O=\beta_{1}*N1+\beta_{2}*N2+\beta_{3}*\text{interaction}$$

$$\text{Nonlinear}\;\text{hypothesis}\;(\text{interaction}\;\text{only}):\;O=\beta_{3}*\text{interaction}$$

The nonlinear GLM models the interaction term, which is calculated by point-wise multiplying N1 by N2, after setting the minimum of both N1 and N2 to zero. The *R*^2^ for each model was recorded and compared across other hypotheses.

#### 2.9.3. Spatial mixture hypothesis

To test the spatial mixture hypotheses, separate GLMs were fit to each vertex timeseries in the region of network overlap using the equation:

$$\text{Spatial}\;\text{mixture}\;\text{hypothesis:}\;O^{v}=\beta_{1}^{v}*N1+\beta_{2}^{v}*N2$$

Here, the indexing of *O on v* refers to the vertex under investigation. To enable direct comparison with other hypotheses, the resulting *R*^*v*^ was averaged across all *O*^2^. The resulting spatial patterns were not further investigated due to poor *R*^2^ performance (see Results, Section 3.6).

## 3. RESULTS

### 3.1. PROFUMO mode maps

**Fig. 3.**
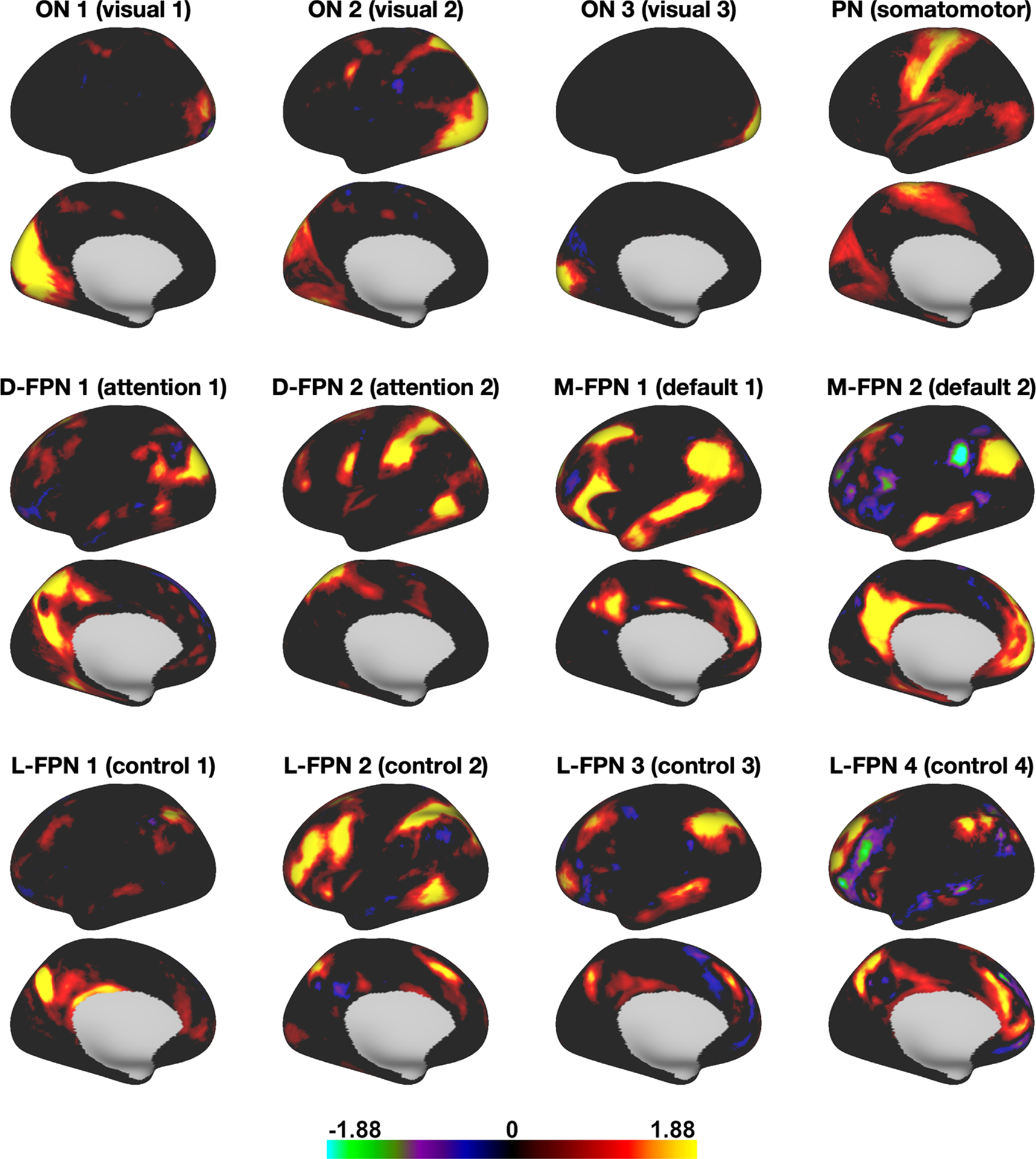
PROFUMO mode (i.e., “network”) maps derived from group PROFUMO using all 12 runs for all 20 participants. For naming purposes, modes were spatially mapped onto the Yeo-7 parcellation (Yeo et al., 2011) and we followed the naming convention suggested in Uddin et al. (2019). FPN = frontoparietal network; L = lateral; D = dorsal; M = medial; ON = occipital network; PN = pericentral network. Figures illustrate networks on the left hemisphere. All 12 networks showed strong bilateral symmetry. Full brain maps can be viewed on Balsa: https://balsa.wustl.edu/study/gm40X.

**Fig. 4.**
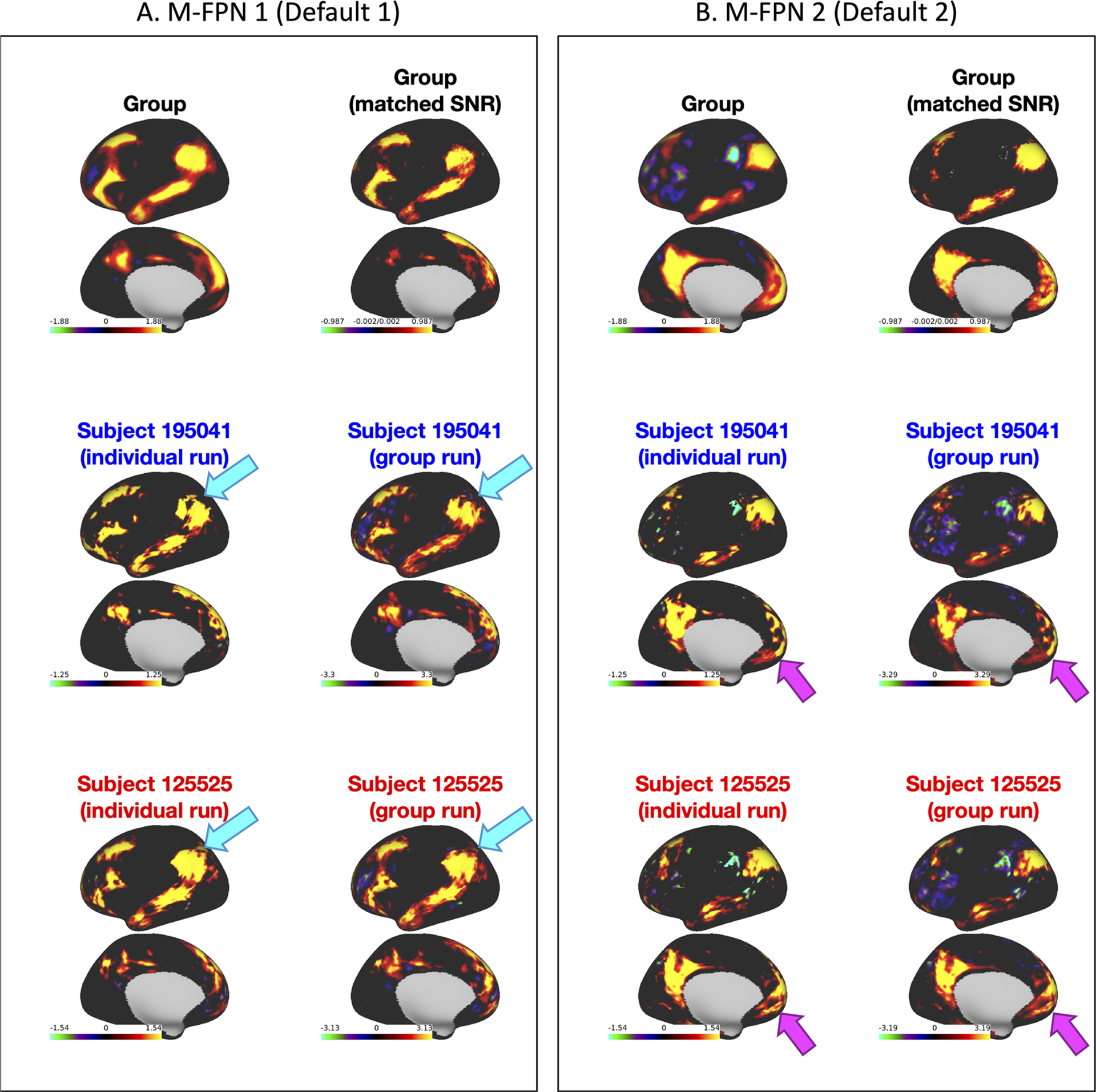
Comparison of two modes across different PROFUMO runs. (A) M-FPN 1 (default 1). (B) M-FPN 2 (default 2). Results display the matched mode from: classic group PROFUMO, classic group PROFUMO using only 12 scans PROFUMO runs, single-subject PROFUMO for two separate example participants, and subject-specific estimates derived from classic group PROFUMO (same two example participants). The example participants were selected to offer a comprehensive overview of the data (including, for example, subjects more similar to and more different to the group). Results for all participants can be viewed on Balsa: https://balsa.wustl.edu/study/gm40X. Results reveal individual differences between single-subject PROFUMO results that accurately match the estimates derived from classic group PROFUMO, confirming that PROFUMO can be used to estimate network organization using only data from a single subject. For example, the cyan arrows in (A) point to a “hole” in the M-FPN 1 network that is consistently observed in subject 195041 and not in subject 125525, and the magenta arrows in (B) point to reproducible but subject-specific frontal patterns in the M-FPN 2 network.

**Fig. 5.**
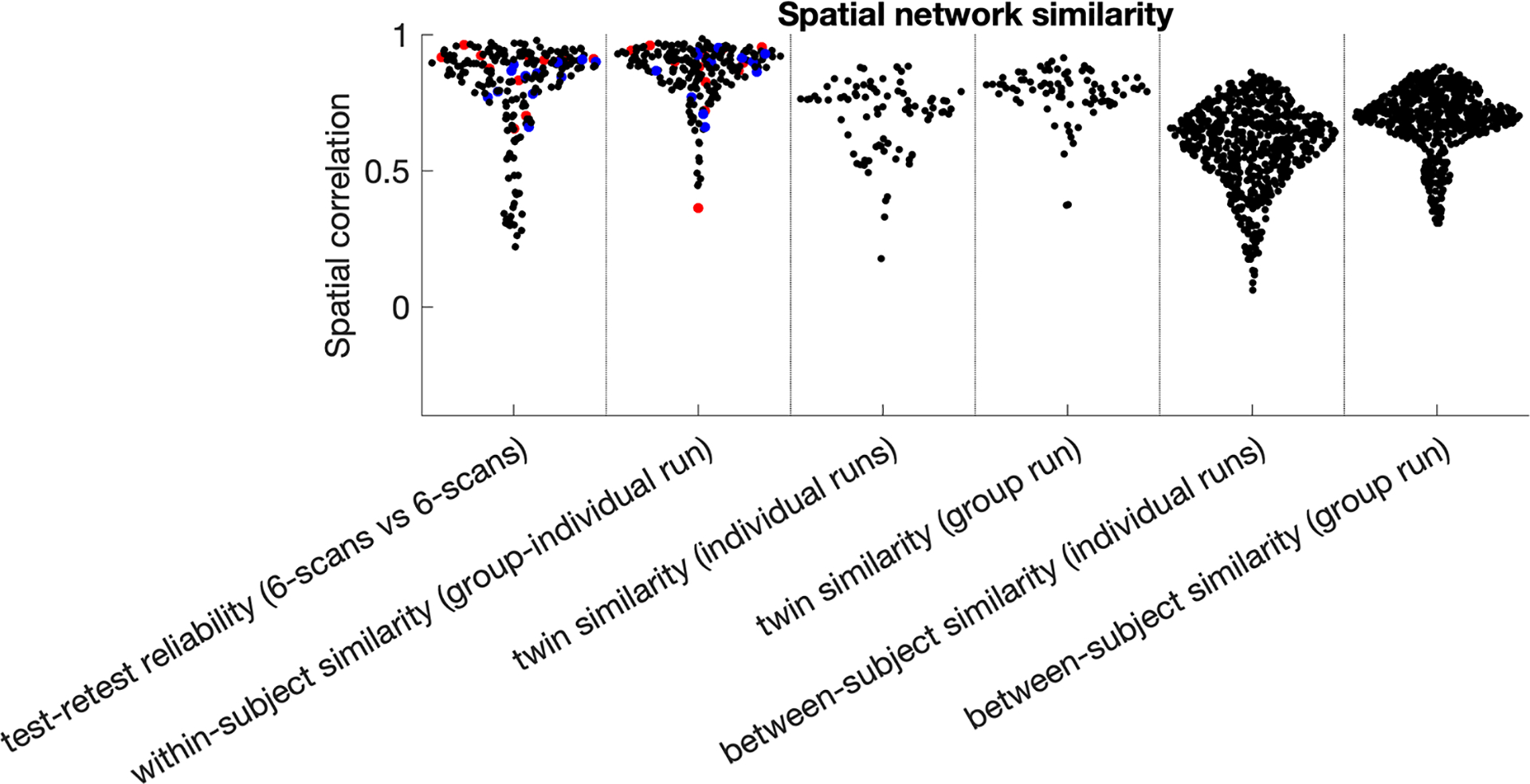
Stability of PROFUMO spatial networks. Results show high within-subject test-retest stability and similarity between individual and group estimates. Similarity within twins is lower than within individuals, but higher than between non-twin participants. Red and blue dots indicate results from the example participants used throughout this paper (separate dots are separate modes).

Out of the total of 20 modes, 12 met the test-retest and subject-group criteria to be considered for further analyses (see Section 2.3). The 12 modes (Fig. 3) covered well-known occipital (visual), pericentral (somatomotor), dorsal frontoparietal (attention), lateral frontoparietal (control), and medial frontoparietal (default) networks (Uddin et al., 2019). These 12 modes were highly replicable across different dimensionalities of PROFUMO (Supplementary Fig. S3). Individual participants had on average 1.4 missing modes (range 0–4; Supplementary Fig. S4). Group maps were stable across the group-PROFUMO using all data and group-PROFUMO using only 12 runs to match subject analyses, albeit with lower weights in the 12-run results reflecting the reduction in SNR (Fig. 4 top row). Mode maps for two example participants derived from single-subject PROFUMO reveal detailed individual specific organization that closely matches maps from the same participants derived from the classic PROFUMO group analysis (Fig. 4 middle and bottom rows). The example participants were chosen as non-twin individuals with a complete set of 12 modes and are representative in terms of all other indices (as shown by highlighting the blue and red data points in Figs. 4, 5, 7 & Supplementary Figs. S4, S6, S7, S8). Subject-specific networks and twin comparisons are shown in Supplementary Figure S5.

### 3.2. PROFUMO mode stability

An initial objective was to determine whether PROFUMO can reliably estimate resting state maps using only data from a single participant. Figure 5 shows that the highest similarity occurs for single-subject PROFUMO spatial maps (test-retest reliability and within-subject similarity in columns 1&2, respectively; mean r = 0.80 ± 0.18); the next highest similarity occurs for twin correlations (Fig. 5 columns 3&4; mean r = 0.70 ± 0.14); and the lowest similarity occurs between non-twin participants (Fig. 5 columns 5&6; mean r = 0.59 ± 0.15). Each similarity pattern shows a “tail” of networks having lower stability (Fig. 5), which on further inspection appeared to be distributed across participants and across networks. When all modes are included (i.e., not only the 12 selected modes and not removing “missing” individual modes), these tails are further expanded but the findings described above regarding comparisons between columns remain evident (see Supplementary Fig. S6). Single-subject PROFUMO spatial maps derived using the 12 runs for the participant (i.e., not including data from other participants) were also highly similar to the estimated subject maps derived from the group data informed by all 20 subjects with 12 runs each (Fig. 5 column 2; mean group-subject r = 0.86 ± 0.11), suggesting reasonable correspondence at least at this dimensionality. Spatial maps estimates informed by group data achieved slightly higher similarity compared to maps estimated from individual runs (Fig. 5 column 4 > 3 and column 6 > 5), revealing the impact of the group prior when performing classic hierarchical PROFUMO. Stability estimates for second-order statistics, including temporal connectivity matrices and spatial overlap matrices, are shown in Supplementary Figures S7 and S8.

### 3.3. Amount of data needed to estimate single-subject modes using PROFUMO

**Fig. 6.**
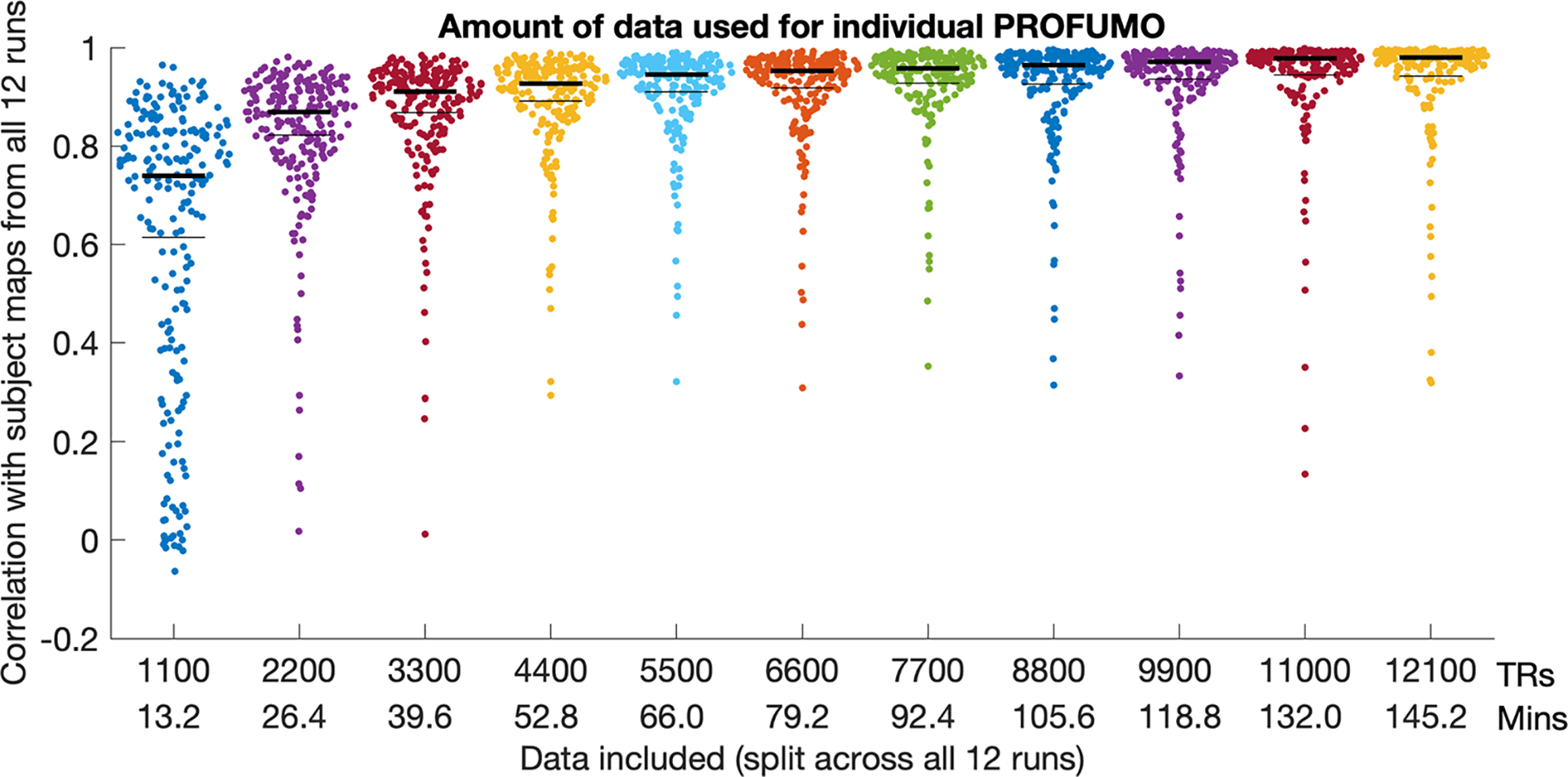
Comparison between subject maps using cut-down versions of the data relative to the subject maps obtained using the full dataset. Each dot represents a single mode-map for a single participant. Thin black lines indicate the mean, and thick black lines indicate the median.

To test how much data from an individual participant were needed to obtain good estimates from single-subject PROFUMO, we systematically varied the number of timepoints (in increments of 1/12th of the run timepoints), and compared the resulting spatial maps to the results that included all timepoints. Mean similarity across the selected (non-missing) mode maps increased when the number of timepoints were increased (Fig. 6) and was near asymptotic above approximately 5000 TRs (approximately 1 hour of data per participant).

### 3.4. Spatial overlap

**Fig. 7.**
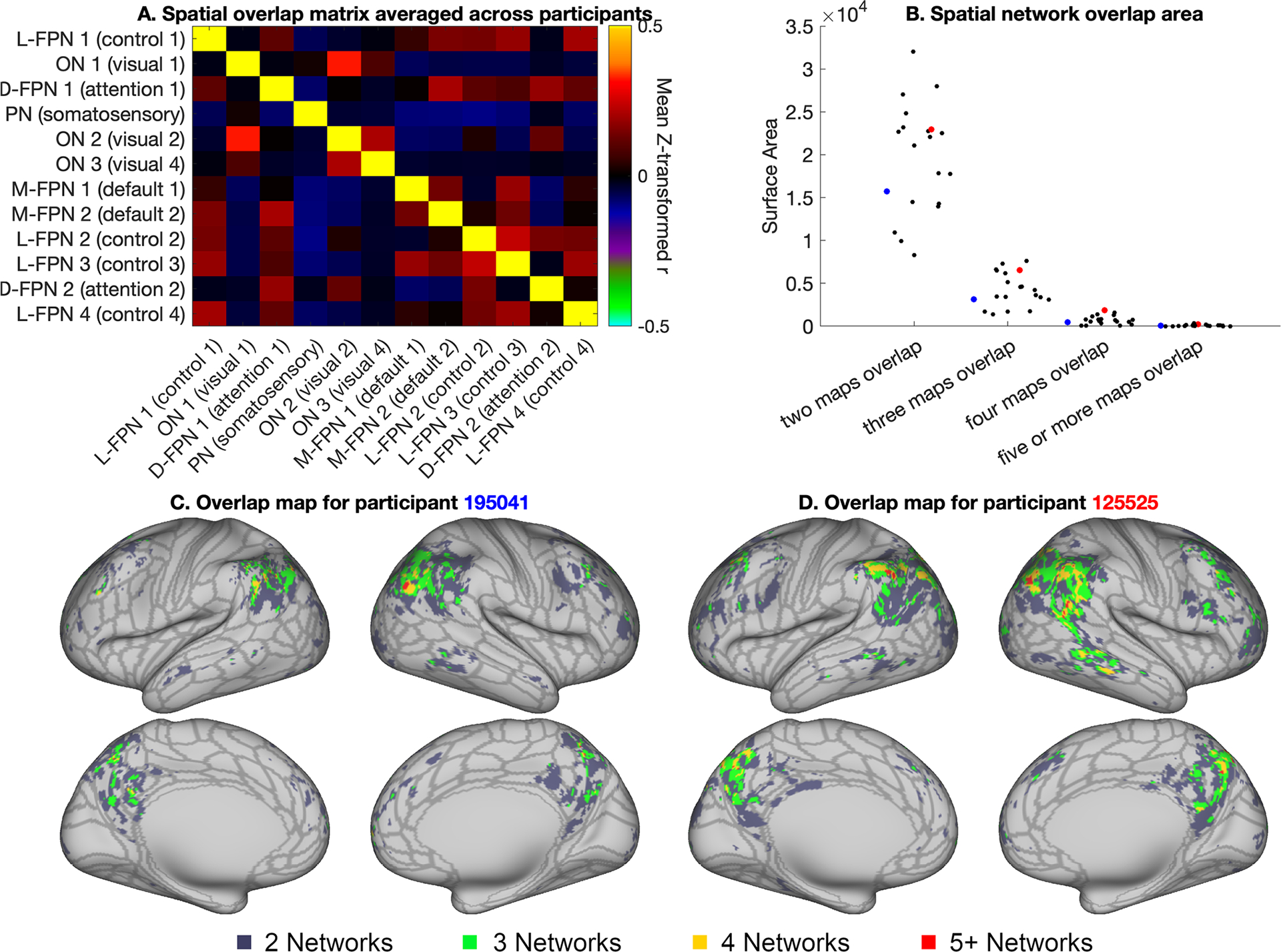
Overview of spatial overlap. (A) Group average spatial overlap matrix, showing pairwise correlations between spatial maps. (B) Number of vertices with 2, 3, 4, 5+ overlapping networks for each individual. Red and blue dots indicate results from the examples participants used throughout this paper. (C and D) Overlap maps for the same two example participants shown in Figure 4 (overlap maps for all participants can be found in Supplementary Fig. S9 and on Balsa: https://balsa.wustl.edu/study/gm40X). For reference, the borders of the HCP_MMP1.0 cortical parcellation from Glasser et al. (2016) are shown in gray in figures C and D.

Consistent with previous work (Bijsterbosch et al., 2019), our results indicate substantial spatial correlation across PROFUMO modes (Fig. 7A). Figure 7A shows the spatial overlap matrix calculated by correlating pairs of weighted PROFUMO group spatial maps. Notably, spatial overlap is ignored in the find-the-biggest overview in Supplementary Figure S5, in which vertices are assigned to a single PROFUMO mode with the highest spatial weight to enable concise visualization of all subject maps. Spatial overlap was primarily localized in the lateral parietal cortex and posterior cingulate -precuneus regions (Fig. 7C, D), consistent with prior work (Bijsterbosch et al., 2019). Spatial overlap maps for two example participants are shown in Figure 7C and D; Supplementary Figure S9 shows maps for all participants and across twin pairs, and Supplementary Figure S1 shows maps for the selected set of 20 2-network overlap pairs. The 2-network spatial area estimates were not associated with DVARS (the temporal derivative of the root mean square variance (Power et al., 2012); r = −0.06, p_uncorrected_ = 0.81), suggesting that spatial overlap was not sensitive to head motion. Conversely, a trend-level association was observed between 5-network spatial area and DVARS (r = −0.45, p_uncorrected_ = 0.05), although we note that our sample size is insufficient to reliably model individual difference (Marek et al., 2022).

### 3.5. Semi-simulated data for 2-network overlap

**Fig. 8.**
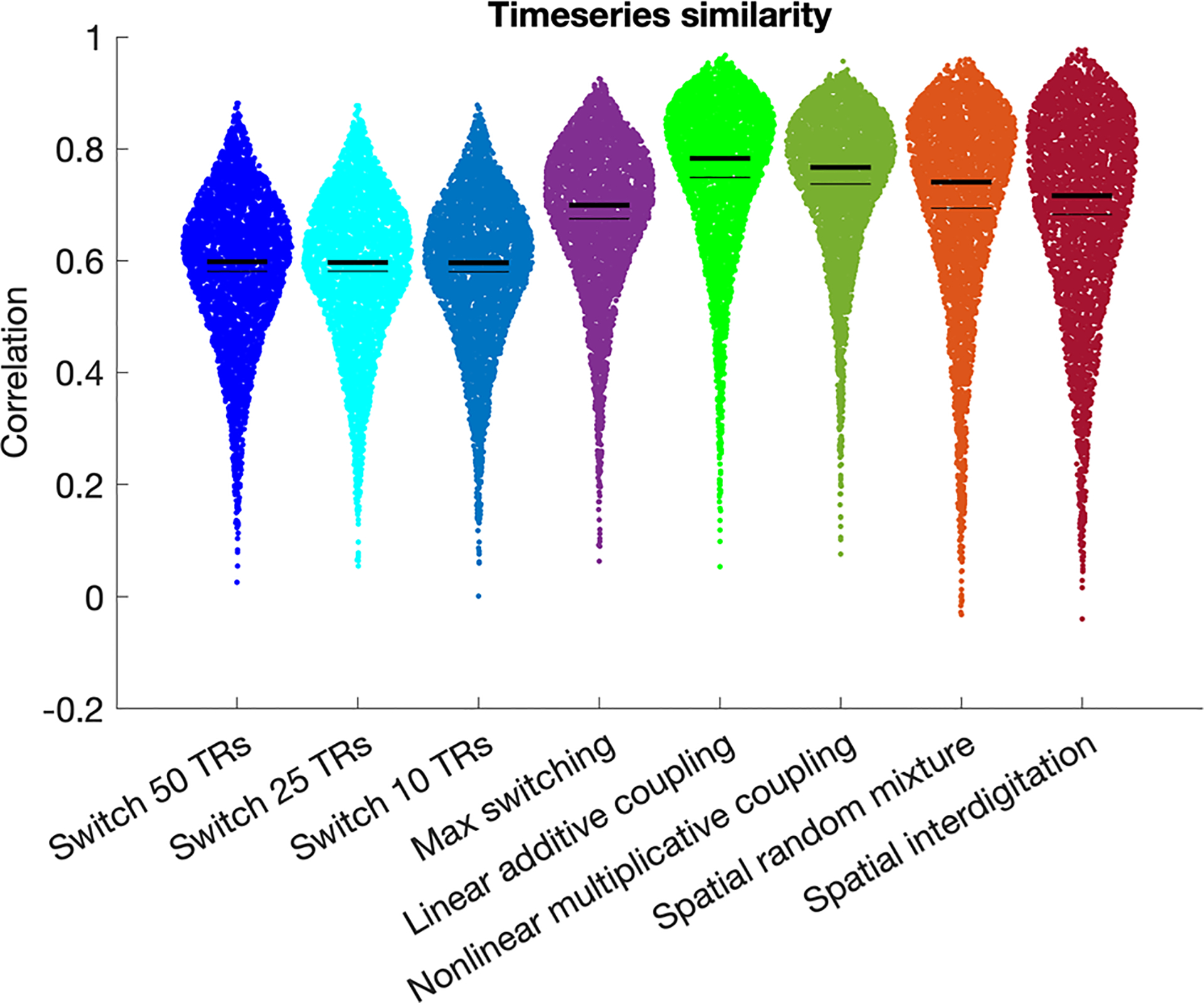
Correlations between the true overlap timeseries and different versions of the semi-simulated overlap timeseries. Thin black lines indicate the mean, and thick black lines indicate the median. Highest similarity was observed for the linear additive coupling semi-simulated overlap timeseries in bright green. Data were combined across all network pairs and all participants; see Supplementary Figure S10 for separate figures per network pair.

We derived semi-simulated versions of the overlap timeseries based on combinations of network 1 and network 2 timeseries (Fig. 2C–E). We assessed how similar the semi-simulated timeseries for the overlap region were, compared to the original overlap timeseries (O) by estimating the Pearson’s correlation coefficient separately for each network pair, each subject, and each run. The highest similarity was observed for the linear additive coupling hypothesis, which achieved a median correlation of 0.783 (Fig. 8). This result was significantly different from the next highest correlation (median of 0.767), observed for the nonlinear multiplicative coupling hypothesis (T = 6.3, p = 3.2*10^−10^, estimated after z-transformation of the correlation values). These findings were observed reliably across all thresholds used for network inclusion and exclusion (see Supplementary Fig. S10).

Across the 20 different network pairs, the linear additive semi-simulated timeseries achieved the highest correlation for 16 network pairs, and the nonlinear multiplicative semi-simulated timeseries achieved the highest correlation for the remaining 4 network pairs (see Supplementary Fig. S11). Notably, each of the 4 network pairs with highest correlations for the nonlinear multiplicative semi-simulated timeseries involved the L-FPN 1 (paired with M-FPN 2, L-FPN 2, L-FPN 3, and L-FPN 4 respectively). The L-FPN 1 network includes a specific subregion (“POS2”) of the parietal-occipital sulcus that is highly distinctive from neighboring regions in its myelin, thickness, connectivity, and task activation (Glasser et al., 2016). As such, the L-FPN 1 network (and POS2 area in particular) warrants future research into their distinctive features, including nonlinear origins of network overlap.

Frequency characteristics were highly similar between the original overlap timeseries and all semi-simulated timeseries (see Supplementary Fig. S12), although spatial interdigitation timeseries exhibited a slightly raised tail and the temporal switching hypothesis (every 10 TRs) has somewhat increased power at 0.1 Hz.

### 3.6. General linear model results

**Fig. 9.**
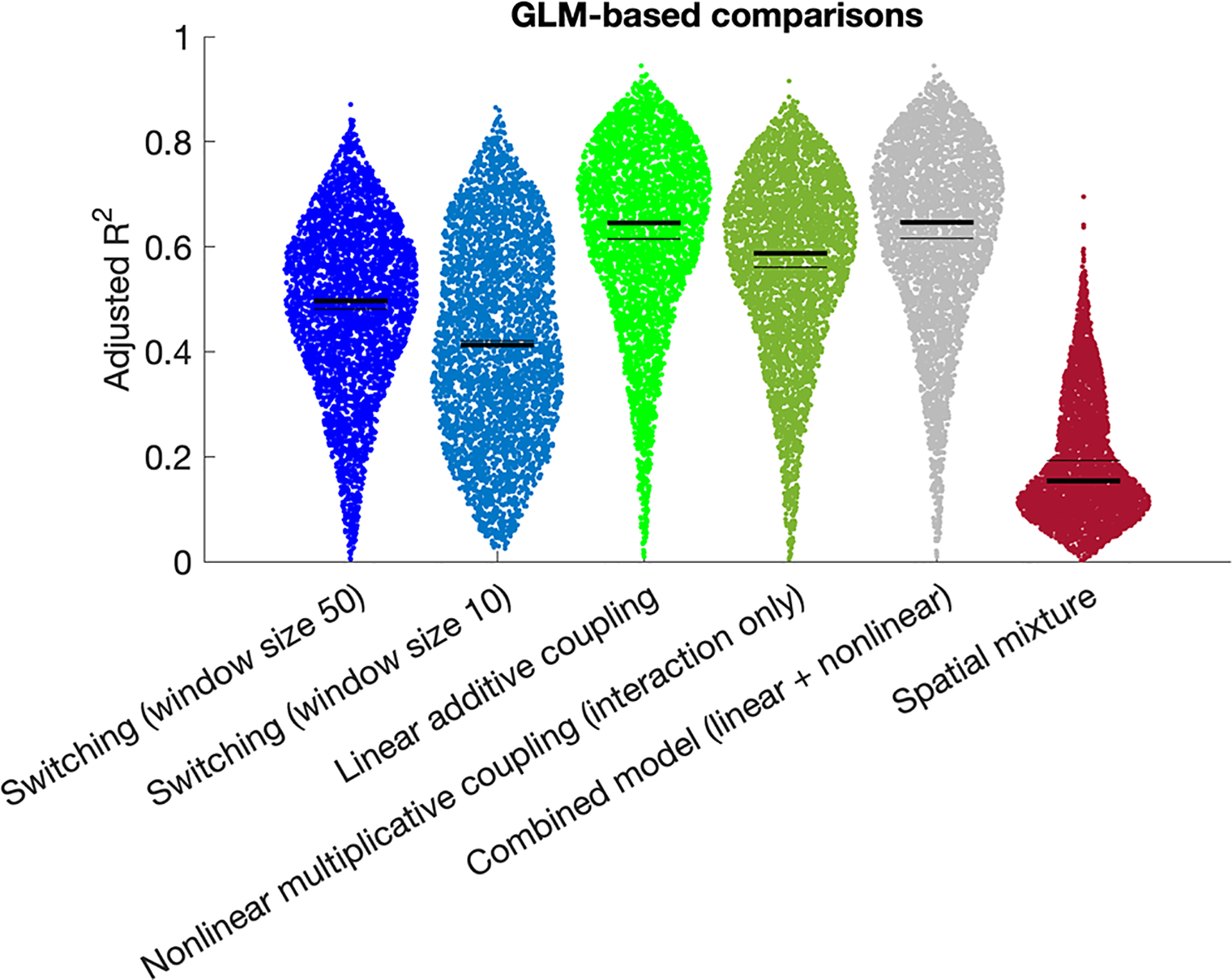
Adjusted *R*^2^ derived from GLM analyses (see Section 2.9) corroborate the findings from our semi-simulated timeseries approach (Fig. 8) showing that the highest model evidence is observed for the linear additive coupling hypothesis (in bright green). Note that this analysis only includes one version of the spatial mixing hypotheses because the data-driven model fit removes the need for alternates.

The results from the semi-simulated timeseries analysis revealed that the linear coupling hypothesis was most strongly supported, followed by the nonlinear coupling hypothesis. We subsequently adopted a GLM-based approach to corroborate these findings. Results confirm that the linear coupling hypothesis achieved the best model fit (median *R*^2^ = 0.65), which was significantly higher than the nonlinear coupling hypothesis (interaction only; median *R*^2^ = 0.59, T = 13.2, p = 4.5*10^−39^; Fig. 9), and was not significantly lower than the combined model with the nonlinear term added (median *R*^2^ = 0.65; T = −0.23, p = 0.82).

## 4. DISCUSSION

Our first aim was to determine whether weighted resting-state spatial networks can be robustly derived from single-subject data without sacrificing correspondence. The results revealed 12 resting-state networks with high test-retest reliability and remarkably good incidental (i.e., non-modeled) correspondence with group-informed network estimates (Fig. 4). Notably, the test-retest reliability of these individual-specific network maps (cosine similarity approximately 0.8 based on Fig. 10A in Harrison et al., 2020) matches or exceeds many previous estimates of reliability of resting-state metrics (e.g., average intraclass correlation 0.29 for temporal functional connectivity from meta analysis across 25 studies; Noble et al., 2019) (Andellini et al., 2015; Braun et al., 2012; Dutt et al., 2022; Fiecas et al., 2013; J. Wang et al., 2017; J.-H. Wang et al., 2011; Liao et al., 2013; Nemani & Lowe, 2021; Noble et al., 2019; Termenon et al., 2016; Yang et al., 2021). These findings support the possibility of future personalized psychiatry approaches where data from an individual would be separately analyzed and compared to a reference cohort to inform clinical decision making. It is possible that increasing the dimensionality to extract more finer-grained resting-state networks (beyond the 12 selected networks out of 20 used here) may reduce test-retest reliability and correspondence. However, we showed high stability of the selected networks across higher dimensionalities of PROFUMO (Supplementary Fig. S1), and previous work in networks defined with independent component analysis reported good reliability up to at least 150 networks (Ma & MacDonald, 2021). Importantly, the functional organization of the brain can meaningfully be studied at multiple levels of complexity along its organizational hierarchy (Bijsterbosch et al., 2021). Here, we specifically chose to investigate the organization of macroscale networks (i.e., low dimensionality) because it describes functional organization in relation to widely studied canonical networks that are consistently observed across datasets, analysis methods, and states (Smith et al., 2009; Uddin et al., 2019; Yeo et al., 2011).

Our second aim was to determine the degree of spatially overlapping network organization. Our findings confirm the presence of extensive and stable network overlap in networks estimated from single-subject data. Patterns of spatially overlapping network organization vary extensively across individuals (Fig. 7 & Supplementary Fig. S6), and previous work has shown that individual differences in these overlap patterns are strongly associated with behavior (Bijsterbosch et al., 2019). As such, the lower dimensional spatial overlap matrix (Fig. 7A) may provide a key summary measure of behaviorally-relevant aspects of spatial organization, while reducing the multiple-comparison burden of vertex-wise analysis of spatial organization.

Spatial overlap between individual-specific weighted resting-state network maps also offers a complementary approach to investigate hub regions. As opposed to traditional hub identification methods that rely on temporal correlations, our weighted network approach emphasizes the role of shared brain regions as part of the spatial organization of functional networks. It is currently unclear whether these distinct temporal versus spatial definitions of hub regions have dissociable or shared neurobiological implications. Some evidence suggests that spatial networks and hubs may maintain functional integrity over time, potentially for homeostatic purposes (Laumann & Snyder, 2021), whereas flexible hubs that can rapidly change their temporal connectivity may provide coordination and switching functions to support cognitive control (Cocuzza et al., 2020; Cole et al., 2013; Gordon et al., 2018). Consistent with this hypothesis, we have shown that spatial overlap was more strongly associated with stable trait-like behavior (Bijsterbosch et al., 2018), whereas temporal correlations tracked transitions between sensorimotor states (Harrison et al., 2020). However, our prior work also suggests that effects of spatial network organization and overlap are observed as temporal correlation estimates when unaccounted for in the parcellation (Bijsterbosch et al., 2018, 2019), which may indicate that the distinction between spatial and temporal hubs could be purely analytical, driven by differences in model cost functions and priors, without distinct neurobiological interpretations. Hence, spatial network overlap and temporally strongly connected nodes may serve distinct functional purposes (e.g., homeostasis versus switching), or may represent alternative analytical estimates of the same underlying neural phenomenon. Further work is needed to gain insight into spatial versus temporal hub definitions and their neurobiological functions.

Our third aim was to systematically test different hypotheses (spatial mixture, dynamic switching, and coupling hypotheses; Fig. 1) regarding the nature of brain regions in which multiple resting-state networks appear to overlap. Our findings supported the linear coupling hypothesis for the 2-network overlap case (Figs. 8 & 9). Future work investigating more complex patterns of overlap across three or more networks might provide support for a different hypothesis. Given that regions of network overlap appear to be actively engaged in processing information from all contributing networks, this suggests that network overlap may play an important role in the integration of information across multiple brain systems. Notably, the results did not conclusively differentiate between linear and nonlinear versions of the coupling hypothesis. This differentiation will be of interest for future research because the linear additive hypothesis may suggest that multiple networks may coexist within overlap regions without influencing one another. Specifically, the combined signal may indicate that both networks behave as they do in non-overlap regions without any cross-network modulation or integration. The nonlinear hypothesis, on the other hand, requires cross-network integration. Prior work has reported that nonlinear binding between multiple task conditions in conjunction hubs (i.e., brain regions that selectively integrate activations) was essential for predicting task activation patterns from functional connectivity data (Ito et al., 2022). Intriguingly, performance for the nonlinear multiplicative hypothesis was not far behind the additive linear hypothesis, which may indicate the presence of network interaction. Although the biological mechanisms of network overlap and additive/multiplicative coupling are unclear, we hypothesize that regions of network overlap do not constitute stand-alone cortical areas but instead play an important role in cross-network integration, for example through the synchronization of local field potentials in band-limited frequency ranges or their enveloppes (Acland et al., 2023).

Despite offering novel insights into weighted resting-state networks estimated within individual participants, this work also has several limitations. First, we investigated network organization at a PROFUMO dimensionality of 20 and investigated 12 resulting networks of high stability. Notably, the 12 networks under investigations were obtained with very high stability across a range of PROFUMO dimensionalities (Supplementary Fig. S1). Nevertheless, network overlap may behave differently at different dimensions of network decomposition, and both higher (Farahibozorg et al., 2021) and lower (Lenzini et al., 2023) network dimensions are of interest for future research. Second, our vertex assignments to network 1, network 2, and overlap regions involved thresholding, which is necessarily a simplification of the weighted network organization. However, the strict threshold for exclusion and inclusion of vertices enforced a relatively conservative definition of overlap that was necessary to make the results interpretable. Importantly, we recommend the use of the unthresholded spatial overlap matrix (Fig. 7A) for brain-behavior investigations of network overlap. Third, the estimation of network overlap, whether based on weighted or thresholded maps, interacts with the contrast-to-noise ratio (CNR) of the data, which varies across the cortex and is higher in the lateral parietal region. As such, potential future efforts to develop a conclusive map of network overlap in the brain should perform careful scaling/normalization to remove any bias resulting from CNR variability across the cortex. Furthermore, individual difference studies should match the amount of data used across individuals to avoid biased estimates of network overlap that may arise from CNR variability across individuals. Fourth, our findings indicating support for the coupling hypothesis are consistent with the underlying outer product model used in PROFUMO (and similar methods such as ICA). In future work, it will be of interest to test mechanistic hypotheses of network overlap using alternative approaches to define the overlap region that do not rely on the outer product model. Notably, alternative tools for the definition of network overlap (such as Karahanoğlu & Van De Ville, 2015; Li et al., 2017) might render different overlap results and might have different reliability and data-needs. Fifth, it remains a possibility that spatial mixing and/or dynamic switching may occur at finer spatial and temporal resolutions that cannot be resolved using resting-state MRI data. Furthermore, it is possible that temporally lagged correlation structure may play a role in regions of spatial overlap, which can be challenging to accurately discern using functional MRI data (Smith et al., 2011) but may be feasible in some situations using deconvolution approaches (Mill et al., 2017). Although beyond the scope of the current paper, we plan to test spatial mixing, dynamic switching, and temporally lagged hypotheses at sub-MRI scales in future research using invasive recording techniques in non-human primates.

Taken together, we showed that weighted resting-state networks derived from single-subject data are stable, correspond closely to group-informed networks, and capture overlapping network organization, and are therefore important targets for clinical biomarker research. We also showed that overlapping network organization is indicative of coupling between networks, providing a mechanistic hypothesis for the functional role of these regions.

## Supplementary Material

Supplementary Material

## Data Availability

The data used in this paper are freely available from https://db.humanconnectome.org/ (requires free registration). All code for this paper is available on GitHub: https://github.com/JanineBijsterbosch/Individual_PROFUMO. Brain maps from Figures 3, 4, and 7 can be viewed in Balsa (including maps for all participants): https://balsa.wustl.edu/study/gm40X.

## References

[R1] Acland BT, Palanca BJA, Bijsterbosch JD, & Snyder LH (2023). The most prominent electrophysiological effect of ketamine is not sufficient to cause anesthesia. In bioRxiv (p. 2023.09.26.559594). 10.1101/2023.09.26.559594

[R2] Andellini M, Cannatà V, Gazzellini S, Bernardi B, & Napolitano A (2015). Test-retest reliability of graph metrics of resting state MRI functional brain networks: A review. Journal of Neuroscience Methods, 253, 183–192. 10.1016/j.jneumeth.2015.05.020 26072249

[R3] Bertolero MA, Yeo BTT, Bassett DS, & D’Esposito M (2018). A mechanistic model of connector hubs, modularity and cognition. Nature Human Behaviour, 2(10), 765–777. 10.1038/s41562-018-0420-6 PMC6322416 30631825

[R4] Bijsterbosch JD, Beckmann CF, Woolrich MW, Smith SM, & Harrison SJ (2019). The relationship between spatial configuration and functional connectivity of brain regions revisited. eLife, 8. 10.7554/eLife.44890 PMC6541435 31066676

[R5] Bijsterbosch JD, Harrison SJ, Jbabdi S, Woolrich M, Beckmann C, Smith S, & Duff EP (2020). Challenges and future directions for representations of functional brain organization. Nature Neuroscience, 1–12. 10.1038/s41593-020-00726-z 33106677

[R6] Bijsterbosch JD, Valk SL, Wang D, & Glasser MF (2021). Recent developments in representations of the connectome. NeuroImage, 243, 118533. 10.1016/j.neuroimage.2021.118533 34469814 PMC8842504

[R7] Bijsterbosch JD, Woolrich MW, Glasser MF, Robinson EC, Beckmann CF, Van Essen DC, Harrison SJ, & Smith SM (2018). The relationship between spatial configuration and functional connectivity of brain regions. eLife, 7. 10.7554/eLife.32992 PMC5860869 29451491

[R8] Blazquez Freches G, Haak KV, Bryant KL, Schurz M, Beckmann CF, & Mars RB (2020). Principles of temporal association cortex organisation as revealed by connectivity gradients. Brain Structure & Function, 225(4), 1245–1260. 10.1007/s00429-020-02047-0 32157450 PMC7270054

[R9] Braga RM, & Buckner RL (2017). Parallel interdigitated distributed networks within the individual estimated by intrinsic functional connectivity. Neuron, 95(2), 457–471. e5. 10.1016/j.neuron.2017.06.038 28728026 PMC5519493

[R10] Braga RM, Van Dijk KRA, Polimeni JR, Eldaief MC, & Buckner RL (2019). Parallel distributed networks resolved at high resolution reveal close juxtaposition of distinct regions. Journal of Neurophysiology, 121(4), 1513–1534. 10.1152/jn.00808.2018 30785825 PMC6485740

[R11] Braun U, Plichta MM, Esslinger C, Sauer C, Haddad L, Grimm O, Mier D, Mohnke S, Heinz A, Erk S, Walter H, Seiferth N, Kirsch P, & Meyer-Lindenberg A (2012). Test–retest reliability of resting-state connectivity network characteristics using fMRI and graph theoretical measures. NeuroImage, 59(2), 1404–1412. 10.1016/j.neuroimage.2011.08.044 21888983

[R12] Buckner RL, Sepulcre J, Talukdar T, Krienen FM, Liu H, Hedden T, Andrews-Hanna JR, Sperling RA, & Johnson KA (2009). Cortical hubs revealed by intrinsic functional connectivity: Mapping, assessment of stability, and relation to Alzheimer’s disease. The Journal of Neuroscience, 29(6), 1860–1873. 10.1523/JNEUROSCI.5062-08.2009 19211893 PMC2750039

[R13] Cocuzza CV, Ito T, Schultz D, Bassett DS, & Cole MW (2020). Flexible coordinator and switcher hubs for adaptive task control. The Journal of Neuroscience, 40(36), 6949–6968. 10.1523/JNEUROSCI.2559-19.2020 32732324 PMC7470914

[R14] Cole MW, Reynolds JR, Power JD, Repovs G, Anticevic A, & Braver TS (2013). Multi-task connectivity reveals flexible hubs for adaptive task control. Nature Neuroscience, 16(9), 1348–1355. 10.1038/nn.3470 23892552 PMC3758404

[R15] Dutt RK, Hannon K, Easley TO, Griffis JC, Zhang W, & Bijsterbosch JD (2022). Mental health in the UK Biobank: A roadmap to self-report measures and neuroimaging correlates. Human Brain Mapping, 43(2), 816–832. 10.1002/hbm.25690 34708477 PMC8720192

[R16] Farahibozorg S-R, Bijsterbosch JD, Gong W, Jbabdi S, Smith SM, Harrison SJ, & Woolrich MW (2021). Hierarchical modelling of functional brain networks in population and individuals from big fMRI data. NeuroImage, 118513. 10.1016/j.neuroimage.2021.118513 34450262 PMC8526871

[R17] Fiecas M, Ombao H, van Lunen D, Baumgartner R, Coimbra A, & Feng D (2013). Quantifying temporal correlations: A test–retest evaluation of functional connectivity in resting-state fMRI. NeuroImage, 65, 231–241. 10.1016/j.neuroimage.2012.09.052 23032492

[R18] Glasser MF, Coalson TS, Robinson EC, Hacker CD, Harwell J, Yacoub E, Ugurbil K, Andersson J, Beckmann CF, Jenkinson M, Smith SM, & Van Essen DC (2016). A multi-modal parcellation of human cerebral cortex. Nature, 536(7615), 171–178. 10.1038/nature18933 27437579 PMC4990127

[R19] Glasser MF, Sotiropoulos SN, Wilson JA, Coalson TS, Fischl B, Andersson JL, Xu J, Jbabdi S, Webster M, Polimeni JR, Van Essen DC, Jenkinson M, & WU-Minn HCP Consortium. (2013). The minimal preprocessing pipelines for the Human Connectome Project. NeuroImage, 80, 105–124. 10.1016/j.neuroimage.2013.04.127 23668970 PMC3720813

[R20] Gordon EM, Laumann TO, Adeyemo B, Gilmore AW, Nelson SM, Dosenbach NUF, & Petersen SE (2017). Individual-specific features of brain systems identified with resting state functional correlations. NeuroImage, 146, 918–939. 10.1016/j.neuroimage.2016.08.032 27640749 PMC5321842

[R21] Gordon EM, Laumann TO, Adeyemo B, & Petersen SE (2017). Individual variability of the system-level organization of the human brain. Cerebral Cortex, 27(1), 386–399. 10.1093/cercor/bhv239 26464473 PMC5939195

[R22] Gordon EM, Laumann TO, Gilmore AW, Newbold DJ, Greene DJ, Berg JJ, Ortega M, Hoyt-Drazen C, Gratton C, Sun H, Hampton JM, Coalson RS, Nguyen AL, McDermott KB, Shimony JS, Snyder AZ, Schlaggar BL, Petersen SE, Nelson SM, & Dosenbach NUF (2017). Precision functional mapping of individual human brains. Neuron, 95(4), 791–807.e7. 10.1016/j.neuron.2017.07.011 28757305 PMC5576360

[R23] Gordon EM, Lynch CJ, Gratton C, Laumann TO, Gilmore AW, Greene DJ, Ortega M, Nguyen AL, Schlaggar BL, Petersen SE, Dosenbach NUF, & Nelson SM (2018). Three distinct sets of connector hubs integrate human brain function. Cell Reports, 24(7), 1687–1695.e4. 10.1016/j.celrep.2018.07.050 30110625 PMC6886580

[R24] Gratton C, Kraus BT, Greene DJ, Gordon EM, Laumann TO, Nelson SM, Dosenbach NUF, & Petersen SE (2020). Defining individual-specific functional neuroanatomy for precision psychiatry. Biological Psychiatry, 88(1), 28–39. 10.1016/j.biopsych.2019.10.026 31916942 PMC7203002

[R25] Gratton C, Laumann TO, Nielsen AN, Greene DJ, Gordon EM, Gilmore AW, Nelson SM, Coalson RS, Snyder AZ, Schlaggar BL, Dosenbach NUF, & Petersen SE (2018). Functional brain networks are dominated by stable group and individual factors, not cognitive or daily variation. Neuron, 98(2), 439–452.e5. 10.1016/j.neuron.2018.03.035 29673485 PMC5912345

[R26] Griffanti L, Salimi-Khorshidi G, Beckmann CF, Auerbach EJ, Douaud G, Sexton CE, Zsoldos E, Ebmeier KP, Filippini N, Mackay CE, Moeller S, Xu J, Yacoub E, Baselli G, Ugurbil K, Miller KL, & Smith SM (2014). ICA-based artefact removal and accelerated fMRI acquisition for improved resting state network imaging. NeuroImage, 95, 232–247. 10.1016/j.neuroimage.2014.03.034 24657355 PMC4154346

[R27] Haak KV, Marquand AF, & Beckmann CF (2018). Connectopic mapping with resting-state fMRI. NeuroImage, 170, 83–94. 10.1016/j.neuroimage.2017.06.075 28666880

[R28] Harrison SJ, Bijsterbosch JD, Segerdahl AR, Fitzgibbon SP, Farahibozorg S-R, Duff EP, Smith SM, & Woolrich MW (2020). Modelling subject variability in the spatial and temporal characteristics of functional modes. NeuroImage, 222, 117226. 10.1016/j.neuroimage.2020.117226 32771617 PMC7779373

[R29] Harrison SJ, Woolrich MW, Robinson EC, Glasser MF, Beckmann CF, Jenkinson M, & Smith SM (2015). Large-scale probabilistic functional modes from resting state fMRI. NeuroImage, 109, 217–231. 10.1016/j.neuroimage.2015.01.013 25598050 PMC4349633

[R30] Hutchison RM, Womelsdorf T, Allen EA, Bandettini PA, Calhoun VD, Corbetta M, Della Penna S, Duyn JH, Glover GH, Gonzalez-Castillo J, Handwerker DA, Keilholz S, Kiviniemi V, Leopold DA, de Pasquale F, Sporns O, Walter M, & Chang C (2013). Dynamic functional connectivity: Promise, issues, and interpretations. NeuroImage, 80, 360–378. 10.1016/j.neuroimage.2013.05.079 23707587 PMC3807588

[R31] Insel TR (2014). The NIMH Research Domain Criteria (RDoC) Project: Precision medicine for psychiatry. The American Journal of Psychiatry, 171(4), 395–397. 10.1176/appi.ajp.2014.14020138 24687194

[R32] Ito T, Yang GR, Laurent P, Schultz DH, & Cole MW (2022). Constructing neural network models from brain data reveals representational transformations linked to adaptive behavior. Nature Communications, 13(1), 673. 10.1038/s41467-022-28323-7 PMC8814166 35115530

[R33] Karahanoğlu FI, & Van De Ville D (2015). Transient brain activity disentangles fMRI resting-state dynamics in terms of spatially and temporally overlapping networks. Nature Communications, 6, 7751. 10.1038/ncomms8751 PMC4518303 26178017

[R34] Kong R, Li J, Orban C, Sabuncu MR, Liu H, Schaefer A, Sun N, Zuo X-N, Holmes AJ, Eickhoff SB, & Yeo BTT (2019). Spatial topography of individual-specific cortical networks predicts human cognition, personality, and emotion. Cerebral Cortex, 29(6), 2533–2551. 10.1093/cercor/bhy123 29878084 PMC6519695

[R35] Kuhn HW (1955). The Hungarian method for the assignment problem. Naval Research Logistics Quarterly, 2(1–2), 83–97. 10.1002/nav.3800020109

[R36] Laumann TO, Gordon EM, Adeyemo B, Snyder AZ, Joo SJ, Chen M-Y, Gilmore AW, McDermott KB, Nelson SM, Dosenbach NUF, Schlaggar BL, Mumford JA, Poldrack RA, & Petersen SE (2015). Functional system and areal organization of a highly sampled individual human brain. Neuron, 87(3), 657–670. 10.1016/j.neuron.2015.06.037 26212711 PMC4642864

[R37] Laumann TO, & Snyder AZ (2021). Brain activity is not only for thinking. Current Opinion in Behavioral Sciences, 40, 130–136. 10.1016/j.cobeha.2021.04.002 PMC13268537 42306170

[R38] Lee K, Lina J-M, Gotman J, & Grova C (2016). SPARK: Sparsity-based analysis of reliable k-hubness and overlapping network structure in brain functional connectivity. NeuroImage, 134, 434–449. 10.1016/j.neuroimage.2016.03.049 27046111

[R39] Lenzini P, Earnest T, Ha SM, Bani A, Sotiras A, & Bijsterbosch J (2023). Morphological versus functional network organization: A comparison between structural covariance networks and probabilistic functional modes. Machine Learning in Clinical Neuroimaging, 163–172. 10.1007/978-3-031-44858-4_16

[R40] Li H, Satterthwaite TD, & Fan Y (2017). Large-scale sparse functional networks from resting state fMRI. NeuroImage, 156, 1–13. 10.1016/j.neuroimage.2017.05.004 28483721 PMC5568802

[R41] Liao X-H, Xia M-R, Xu T, Dai Z-J, Cao X-Y, Niu H-J, Zuo X-N, Zang Y-F, & He Y (2013). Functional brain hubs and their test–retest reliability: A multiband resting-state functional MRI study. NeuroImage, 83, 969–982. 10.1016/j.neuroimage.2013.07.058 23899725

[R42] Lin Y, Ma J, Gu Y, Yang S, Li LMW, & Dai Z (2018). Intrinsic overlapping modular organization of human brain functional networks revealed by a multiobjective evolutionary algorithm. NeuroImage, 181, 430–445. 10.1016/j.neuroimage.2018.07.019 30005918

[R43] Ma Y, & MacDonald AW Iii. (2021). Impact of independent component analysis dimensionality on the test-retest reliability of resting-state functional connectivity. Brain Connectivity, 11(10), 875–886. 10.1089/brain.2020.0970 33926215 PMC8817714

[R44] Marek S, Tervo-Clemmens B, Calabro FJ, Montez DF, Kay BP, Hatoum AS, Donohue MR, Foran W, Miller RL, Hendrickson TJ, Malone SM, Kandala S, Feczko E, Miranda-Dominguez O, Graham AM, Earl EA, Perrone AJ, Cordova M, Doyle O, … Dosenbach NUF (2022). Reproducible brain-wide association studies require thousands of individuals. Nature, 603(7902), 654–660. 10.1038/s41586-022-04492-9 35296861 PMC8991999

[R45] Mill RD, Bagic A, Bostan A, Schneider W, & Cole MW (2017). Empirical validation of directed functional connectivity. NeuroImage, 146, 275–287. 10.1016/j.neuroimage.2016.11.037 27856312 PMC5321749

[R46] Munkres J (1957). Algorithms for the assignment and transportation problems. Journal of the Society for Industrial and Applied Mathematics, 5(1), 32–38. 10.1137/0105003

[R47] Najafi M, McMenamin BW, Simon JZ, & Pessoa L (2016). Overlapping communities reveal rich structure in large-scale brain networks during rest and task conditions. NeuroImage, 135, 92–106. 10.1016/j.neuroimage.2016.04.054 27129758 PMC4915991

[R48] Nemani A, & Lowe MJ (2021). Seed-based test-retest reliability of resting state functional magnetic resonance imaging at 3 T and 7 T. Medical Physics, 48(10), 5756–5764. 10.1002/mp.15210 34486120

[R49] Noble S, Scheinost D, & Constable RT (2019). A decade of test-retest reliability of functional connectivity: A systematic review and meta-analysis. NeuroImage, 203, 116157. 10.1016/j.neuroimage.2019.116157 31494250 PMC6907736

[R50] Nunnally JC (1967). Psychometric theory (Vol. 640). McGraw-Hill Psychometric theory.

[R51] Poldrack RA (2017). Precision neuroscience: Dense sampling of individual brains. Neuron, 95(4), 727–729. 10.1016/j.neuron.2017.08.002 28817793

[R52] Poldrack RA, Laumann TO, Koyejo O, Gregory B, Hover A, Chen M-Y, Gorgolewski KJ, Luci J, Joo SJ, Boyd RL, Hunicke-Smith S, Simpson ZB, Caven T, Sochat V, Shine JM, Gordon E, Snyder AZ, Adeyemo B, Petersen SE, … Mumford JA (2015). Long-term neural and physiological phenotyping of a single human. Nature Communications, 6, 8885. 10.1038/ncomms9885 PMC4682164 26648521

[R53] Power JD, Barnes KA, Snyder AZ, Schlaggar BL, & Petersen SE (2012). Spurious but systematic correlations in functional connectivity MRI networks arise from subject motion. NeuroImage, 59(3), 2142–2154. 10.1016/j.neuroimage.2011.10.018 22019881 PMC3254728

[R54] Robinson EC, Jbabdi S, Glasser MF, Andersson J, Burgess GC, Harms MP, Smith SM, Van Essen DC, & Jenkinson M (2014). MSM: A new flexible framework for Multimodal Surface Matching. NeuroImage, 100, 414–426. 10.1016/j.neuroimage.2014.05.069 24939340 PMC4190319

[R55] Salimi-Khorshidi G, Douaud G, Beckmann CF, Glasser MF, Griffanti L, & Smith SM (2014). Automatic denoising of functional MRI data: Combining independent component analysis and hierarchical fusion of classifiers. NeuroImage, 90, 449–468. 10.1016/j.neuroimage.2013.11.046 24389422 PMC4019210

[R56] Smith SM, Beckmann CF, Andersson J, Auerbach EJ, Bijsterbosch J, Douaud G, Duff E, Feinberg DA, Griffanti L, Harms MP, Kelly M, Laumann T, Miller KL, Moeller S, Petersen S, Power J, Salimi-Khorshidi G, Snyder AZ, Vu AT, … WU-Minn HCP Consortium. (2013). Resting-state fMRI in the Human Connectome Project. NeuroImage, 80, 144–168. 10.1016/j.neuroimage.2013.05.039 23702415 PMC3720828

[R57] Smith SM, Fox PT, Miller KL, Glahn DC, Fox PM, Mackay CE, Filippini N, Watkins KE, Toro R, Laird AR, & Beckmann CF (2009). Correspondence of the brain’s functional architecture during activation and rest. Proceedings of the National Academy of Sciences of the United States of America, 106(31), 13040–13045. 10.1073/pnas.0905267106 19620724 PMC2722273

[R58] Smith SM, Miller KL, Salimi-Khorshidi G, Webster M, Beckmann CF, Nichols TE, Ramsey JD, & Woolrich MW (2011). Network modelling methods for FMRI. NeuroImage, 54(2), 875–891. 10.1016/j.neuroimage.2010.08.063 20817103

[R59] Termenon M, Jaillard A, Delon-Martin C, & Achard S (2016). Reliability of graph analysis of resting state fMRI using test-retest dataset from the Human Connectome Project. NeuroImage, 142, 172–187. 10.1016/j.neuroimage.2016.05.062 27282475

[R60] Uddin LQ, Yeo BTT, & Spreng RN (2019). Towards a universal taxonomy of macro-scale functional human brain networks. Brain Topography. 10.1007/s10548-019-00744-6 PMC7325607 31707621

[R61] Van Essen DC, Smith SM, Barch DM, Behrens TEJ, Yacoub E, Ugurbil K, & WU-Minn HCP Consortium. (2013). The WU-Minn Human Connectome Project: An overview. NeuroImage, 80(0), 62–79. 10.1016/j.neuroimage.2013.05.041 23684880 PMC3724347

[R62] Vu AT, Jamison K, Glasser MF, Smith SM, Coalson T, Moeller S, Auerbach EJ, Uğurbil K, & Yacoub E (2017). Tradeoffs in pushing the spatial resolution of fMRI for the 7 T Human Connectome Project. NeuroImage, 154, 23–32. 10.1016/j.neuroimage.2016.11.049 27894889 PMC5445004

[R63] Wang D, Buckner RL, Fox MD, Holt DJ, Holmes AJ, Stoecklein S, Langs G, Pan R, Qian T, Li K, Baker JT, Stufflebeam SM, Wang K, Wang X, Hong B, & Liu H (2015). Parcellating cortical functional networks in individuals. Nature Neuroscience, 18(12), 1853–1860. 10.1038/nn.4164 26551545 PMC4661084

[R64] Wang J, Han J, Nguyen VT, Guo L, & Guo CC (2017). Improving the test-retest reliability of resting state fMRI by removing the impact of sleep. Frontiers in Neuroscience, 11, 249. 10.3389/fnins.2017.00249 28533739 PMC5420587

[R65] Wang J-H, Zuo X-N, Gohel S, Milham MP, Biswal BB, & He Y (2011). Graph theoretical analysis of functional brain networks: Test-retest evaluation on short-and long-term resting-state functional MRI data. PLoS One, 6(7), e21976. 10.1371/journal.pone.0021976 21818285 PMC3139595

[R66] Warren DE, Power JD, Bruss J, Denburg NL, Waldron EJ, Sun H, Petersen SE, & Tranel D (2014). Network measures predict neuropsychological outcome after brain injury. Proceedings of the National Academy of Sciences of the United States of America, 111(39), 14247–14252. 10.1073/pnas.1322173111 25225403 PMC4191760

[R67] Williams LM (2016). Precision psychiatry: A neural circuit taxonomy for depression and anxiety. The Lancet Psychiatry, 3(5), 472–480. 10.1016/S2215-0366(15)00579-9 27150382 PMC4922884

[R68] Yang L, Wei J, Li Y, Wang B, Guo H, Yang Y, & Xiang J (2021). Test-retest reliability of synchrony and metastability in resting state fMRI. Brain Sciences, 12(1). 10.3390/brainsci12010066 PMC8773904 35053813

[R69] Yeo BTT, Krienen FM, Sepulcre J, Sabuncu MR, Lashkari D, Hollinshead M, Roffman JL, Smoller JW, Zöllei L, Polimeni JR, Fischl B, Liu H, & Buckner RL (2011). The organization of the human cerebral cortex estimated by intrinsic functional connectivity. Journal of Neurophysiology, 106(3), 1125–1165. 10.1152/jn.00338.2011 21653723 PMC3174820

