# Supplementary material for: Evaluating functional brain organization in individuals and identifying contributions to network overlap
Source: Imaging Neurosci (Camb). 2023 Dec 8;1:imag-1-00046. doi: 10.1162/imag_a_00046 (PMC11867625; doi:10.1162/imag_a_00046)
Supplement: Supplementary Material [file imag_a_00046-supp.pdf]

Supplementary Information

Table S1: PROFUMO model details at the group level

Group Spatial Model	Group signal component	μ_{vm}<br>$p(\mu_{vm} \mid \rho_{vm} = 2) = \mathcal{N}(\mu_{vm} \mid \tau_{\mu 2}, {\gamma_{\mu 2}}^2)$<br>$p(\mu_{vm} \mid \rho_{vm} = 1) = \mathcal{N}(\mu_{vm} \mid \tau_{\mu 1}, {\gamma_{\mu 1}}^2)$<br>$p(\mu_{vm} \mid \rho_{vm} = 0) = \delta(\mu_{vm})$<br>$p(\rho_{vm}) = \prod_{i \in \{0,1,2\}} (\lambda_{\mu i})^{[\rho_{vm} = i]}$	Hyperprior: mixture model prior over group means (inspired by spike-slab distribution to allow sparsity, but modeled as one delta and two Gaussians to allow heavier tails)<br>Hyperparameters of first Gaussian:<br>[illegible]$_{\mu 1}$ and $\gamma^2_{\mu 1}$<br>Hyperparameters of second Gaussian: [illegible]$_{\mu 2}$ and $\gamma^2_{\mu 2}$
	Standard deviation of signal component	σ_{vm}<br>$p(\sigma_{vm}) = \Gamma(\sigma_{vm}^{-2} \mid a_\sigma, b_\sigma)$	Hyperprior: inverse-Gamma distribution, with hyperparameters a_σ and b_σ
	Standard deviation of noise component	$\eta^{(s)}_m \zeta_v$<br>$p(\zeta_v) = \Gamma(\zeta_v^{-2} \mid a_\zeta, b_\zeta)$	Hyperprior: inverse-gamma distribution on ζ_v - defined per voxel, with hyperparameters<br>a_ζ and b_ζ<br>See subject-level table for details of $\eta^{(s)}_m$
	Probability that a given weight is drawn from signal rather than noise distribution	π_{vm}<br>$p(\pi_{vm}) = \beta(\pi_{vm} \mid a_\pi, b_\pi)$	Hyperprior: Beta distribution with hyperparameters<br>a_π and b_π
Group Temporal Model	Temporal precision matrix to encourage consistency across subjects/runs	β<br>$p(\boldsymbol{\beta}) = \mathscr{W}(\boldsymbol{\beta} \mid a_\beta, \boldsymbol{B}_\beta)$	Hyperprior: Wishart distribution with hyper parameters a_β and B_β
Group Amplitude Model	Mean of amplitudes	μ_h<br>$p(\boldsymbol{\mu_h}) = \prod_{m=1}^{M} \mathcal{N}((\boldsymbol{\mu_h})_m \mid \tau_{\boldsymbol{\mu_h}}, \gamma^2_{\boldsymbol{\mu_h}})$	Hyperprior: Gaussian distribution with hyperparameters [illegible]$_{\mu_h}$<br>and $\gamma^2_{\mu_h}$
	Covariance of amplitudes	Σ_h<br>$p(\boldsymbol{\Sigma_h}) = \mathscr{W}(\boldsymbol{\Sigma_h}^{-1} \mid a_h, \boldsymbol{B_h})$	Hyperprior: Wishart distribution with hyperparameters a_h and B_h
Noise Model	Noise does not need group level modeling. This refers to residuals in matrix factorisation done at subject-level only:<br>$p\left(\boldsymbol{D}^{(sr)} - \boldsymbol{P}^{(s)} \boldsymbol{H}^{(sr)} \boldsymbol{A}^{(sr)}\right)$		

Table S2: PROFUMO model details at the subject level

Matrix Factorization	$\boldsymbol{D}^{sr} = \boldsymbol{P}^{s}\boldsymbol{H}^{sr}\boldsymbol{A}^{sr} + \boldsymbol{\varepsilon}^{sr}$ $\quad \boldsymbol{D}^{sr} \in \mathbb{R}^{N_v \times N_t}, \boldsymbol{P}^{s} \in \mathbb{R}^{N_v \times N_m}, \boldsymbol{H}^{sr} \in \mathbb{R}^{N_m \times N_m}, \boldsymbol{A}^{sr} \in \mathbb{R}^{N_m \times N_t}$		
Subject Spatial Model	Subject signal component<br>(first component of a Double Gaussian Mixture Model)	$P^{(s)}_{vm}\,(\vert q^{(s)}_{vm} = 1)$<br>$p\left(\boldsymbol{P}^{(s)}_{vm} \middle\vert q^{(s)}_{vm} = 1\right) = \mathcal{N}\left(\boldsymbol{P}^{(s)}_{vm} \middle\vert \mu_{vm}, \sigma^2_{vm}\right)$	Hyperprior: Gaussian distribution with hyperparameters μ_{vm} and σ_{vm} fed from the group model
	Subject noise component<br>(second component of a Double Gaussian Mixture Model)	$P^{(s)}_{vm}\,(\vert q^{(s)}_{vm} = 0)$<br>$p\left(\boldsymbol{P}^{(s)}_{vm} \middle\vert q^{(s)}_{vm} = 0\right) = \mathcal{N}\left(\boldsymbol{P}^{(s)}_{vm} \middle\vert 0, \left(\eta^{(s)}_{m}\right)^2 \zeta^2_v\right)$	Hyperprior: Zero-mean Gaussian distribution with variance hyperparameters<br>ζ_v fed from the group model; $\eta^{(s)}_m$ described below.
	Binary variable indicating whether a voxel belongs to the signal vs noise component.	$q^{(s)}_{vm}$<br>$p\left(q^{(s)}_{vm}\right) = (\pi_{vm})^{q^{(s)}_{vm}} (1 - \pi_{vm})^{1 - q^{(s)}_{vm}}$	Hyperparameter π_{vm} fed from group membership probability
	Extra parameter to capture variations in noise levels across voxels and modes	$\eta^{(s)}_m$<br>$p\left(\eta^{(s)}_m\right) = \mathcal{N}\left(\eta^{(s)}_m \middle\vert 0, \gamma^2_\eta\right)$	Hyperprior: zero-mean Gaussian distribution with variance γ^2_η
Subject Temporal Model	Signal Timecourse (per mode mode per subject per run)<br>Signal timecourse modeling is informed be HRF-induced autocorrelation structure (K) and between-mode precision matrix α	$A^{(sr)}_m$<br>$p\left(\boldsymbol{A}^{(sr)}_m \middle\vert \alpha\right) = \mathcal{N}\left(\boldsymbol{A}^{(sr)}_m \middle\vert \boldsymbol{0}, \alpha^{-1}\boldsymbol{K_A}\right)$	Hyperprior: Gaussian distribution, with mean 0 and variance $\alpha^{-1}K_A$
	Noise Timecourse (per mode mode per subject per run per timepoint)	$\xi^{(sr)}_{mt}$<br>$p\left(\boldsymbol{\xi}^{(sr)}_{mt} \middle\vert \omega^{(sr)}_m\right) = \mathcal{N}\left(\boldsymbol{\xi}^{(sr)}_{mt} \middle\vert 0, {\omega^{(sr)}_m}^{-1}\right)$<br>$p\left(\omega^{(sr)}_m\right) = \Gamma\left(\omega^{(sr)}_m \middle\vert a_\omega, b_\omega\right)$	Drawn from zero-mean, white Gaussian noise. Gamma hyperprior on noise precision (ω) with hyperparameters a_ω and b_ω
	Temporal precision matrix (per subject, can also be defined per run). This combines the HRF-induced autocorrelation structure (K_B) with a prior on the between-mode precision matrix $\alpha^{(sr)}$<br>See Harrison et al., 2015 for details of a canonical double-gamma HRF modeling in PROFUMO.	$B^{(sr)}$<br>$p\left(\boldsymbol{B}^{(sr)} \middle\vert \boldsymbol{\alpha}^{(sr)}\right) = \mathcal{MN}\left(\boldsymbol{B}^{(sr)} \middle\vert \boldsymbol{0}, {\boldsymbol{\alpha}^{(sr)}}^{-1}, \boldsymbol{K_B}\right)$<br>$p\left(\boldsymbol{\alpha}^{(sr)} \middle\vert \boldsymbol{\beta}\right) = \mathcal{W}\left(\boldsymbol{\alpha}^{(sr)} \middle\vert a_{\boldsymbol{\alpha}}, \boldsymbol{\beta}\right)$	Hyperprior for B: multivariate normal distribution with hyperparameters 0, $\alpha^{(sr)}$ and K_B<br>Hyperprior for $\alpha^{(sr)}$: Wishart distribution with hyperparameters a_α and β, the latter is fed from the group model.
Subject Amplitude Model	Subject- and run-specific amplitudes	$H^{(sr)} = diag(h^{(sr)})$<br>$p\left(\boldsymbol{h}^{(sr)}_m \middle\vert \boldsymbol{\mu_h}, \boldsymbol{\Sigma_h}\right) = \mathcal{N}\left(\boldsymbol{h}^{(sr)}_m \middle\vert \boldsymbol{\mu_h}, \boldsymbol{\Sigma_h}\right)$	Hyperprior: Gaussian distribution with hyperparameters μ_h and Σ_h fed from the group
Subject Noise Model	Noise model to ensure that noise variance is the same in every voxel	$\epsilon^{(sr)}$<br>$p\left(\varepsilon^{(sr)}\right) = \mathcal{MN}\left(\varepsilon^{(sr)} \middle\vert \boldsymbol{0}, \left(\psi^{(sr)}\right)^{-1}\boldsymbol{I}_V, \boldsymbol{I}_T\right)$<br>$= p\left(\boldsymbol{D}^{(sr)} - \boldsymbol{P}^{(s)}\boldsymbol{H}^{(sr)}\boldsymbol{A}^{(sr)}\right)$<br>$p\left(\psi^{(sr)}\right) = \Gamma\left(\psi^{(sr)} \middle\vert a_\psi, b_\psi\right)$	Zero mean, white Gaussian noise. Gamma hyperprior on noise precision with hyperparameters<br>a_ψ and b_ψ

Figure S1: Patterns of network overlap for all 20 network pairs

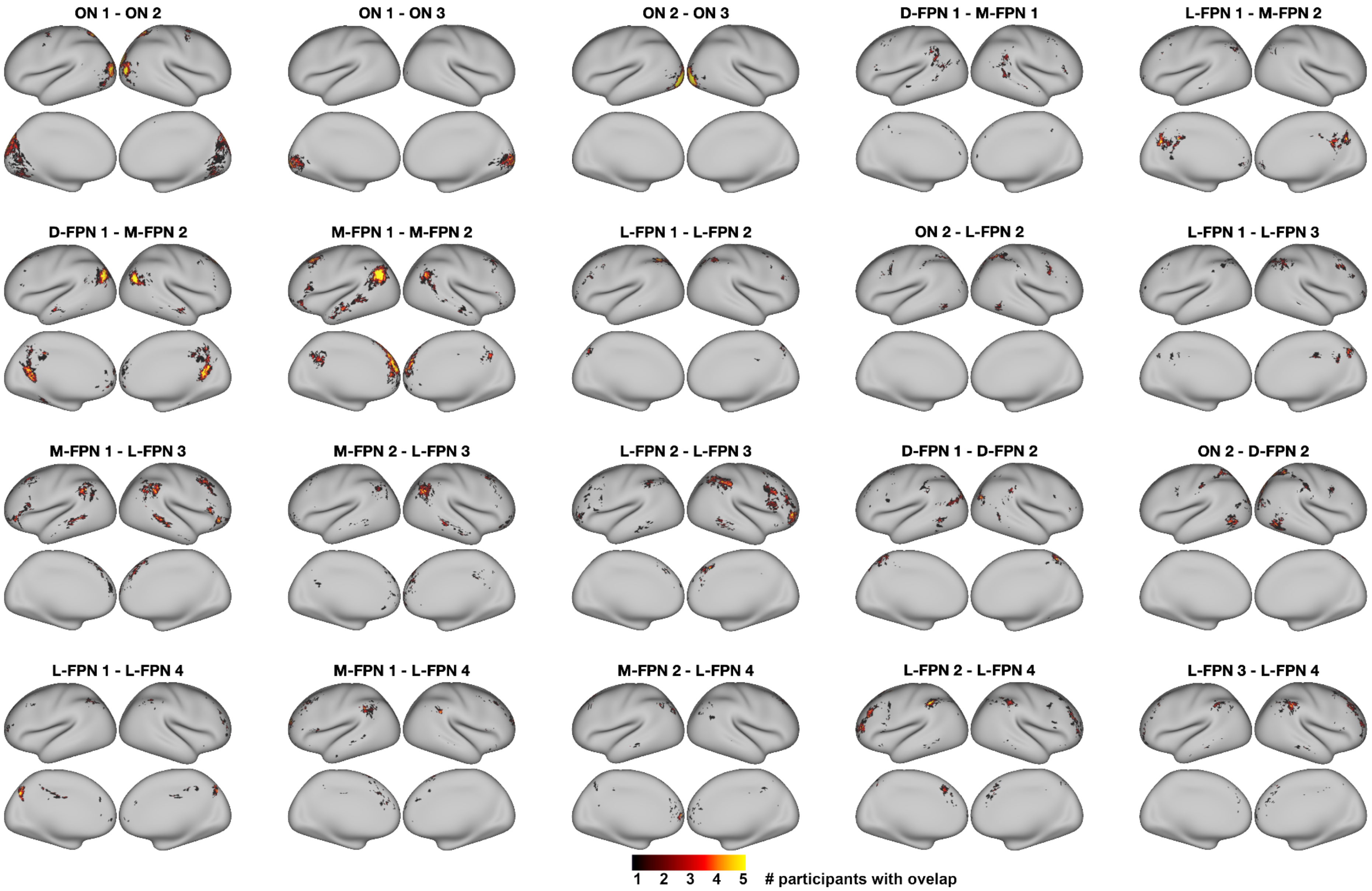

Brain regions of spatial overlap between all 20 2-network pairs are displayed. At each vertex, the value represents the number of participants with overlap.

Figure S2: Interdigitation patterns for all 20 network pairs in example subject 195041

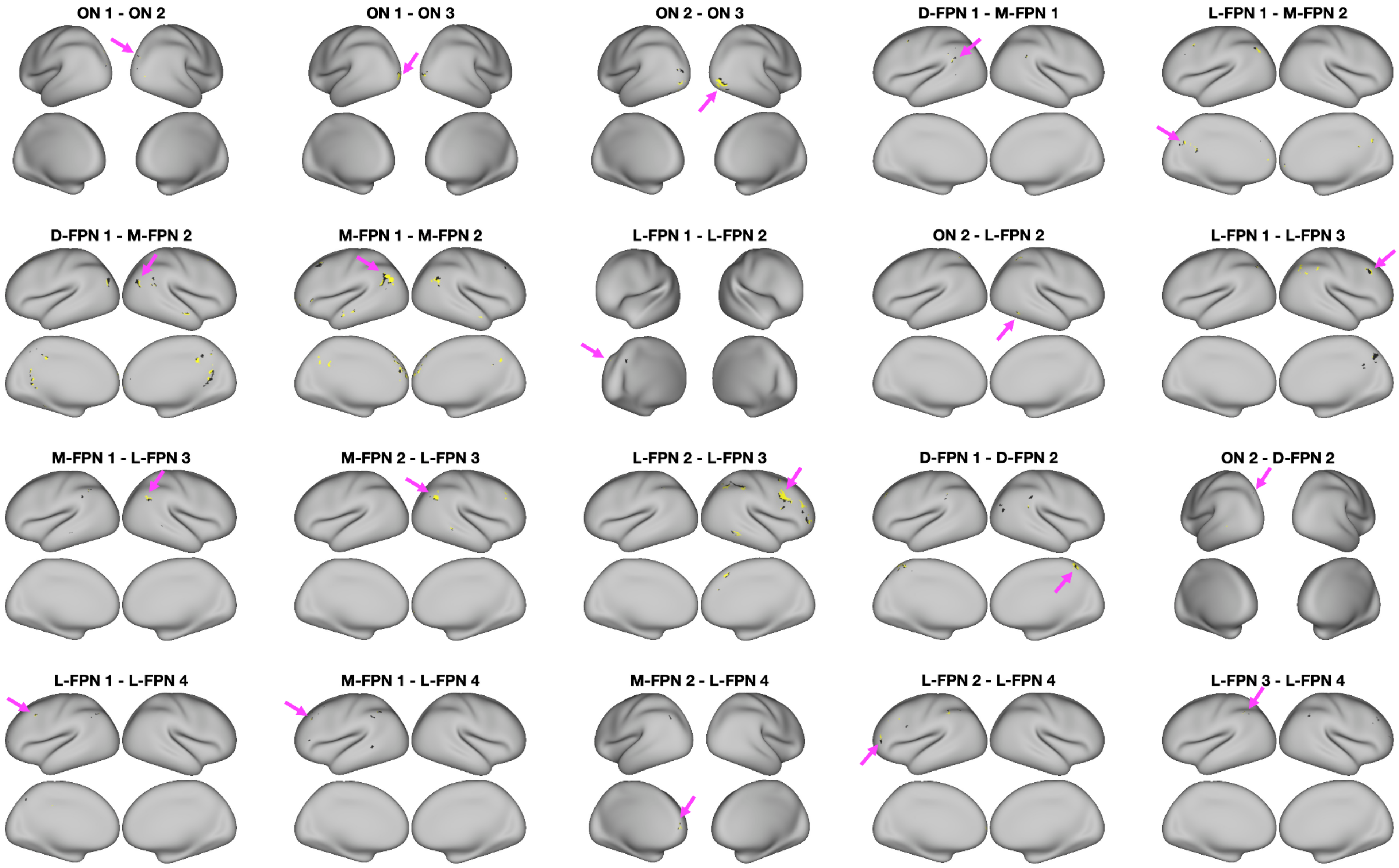

Vertex assignments between network 1 (black) and network 2 (yellow) for all network pairs. Results for subject 195041 are shown as an example.
The arrows were added to assist the reader in locating some of the areas of overlap, because they can be difficult to spot.

Figure S3: Mode stability across dimensionalities

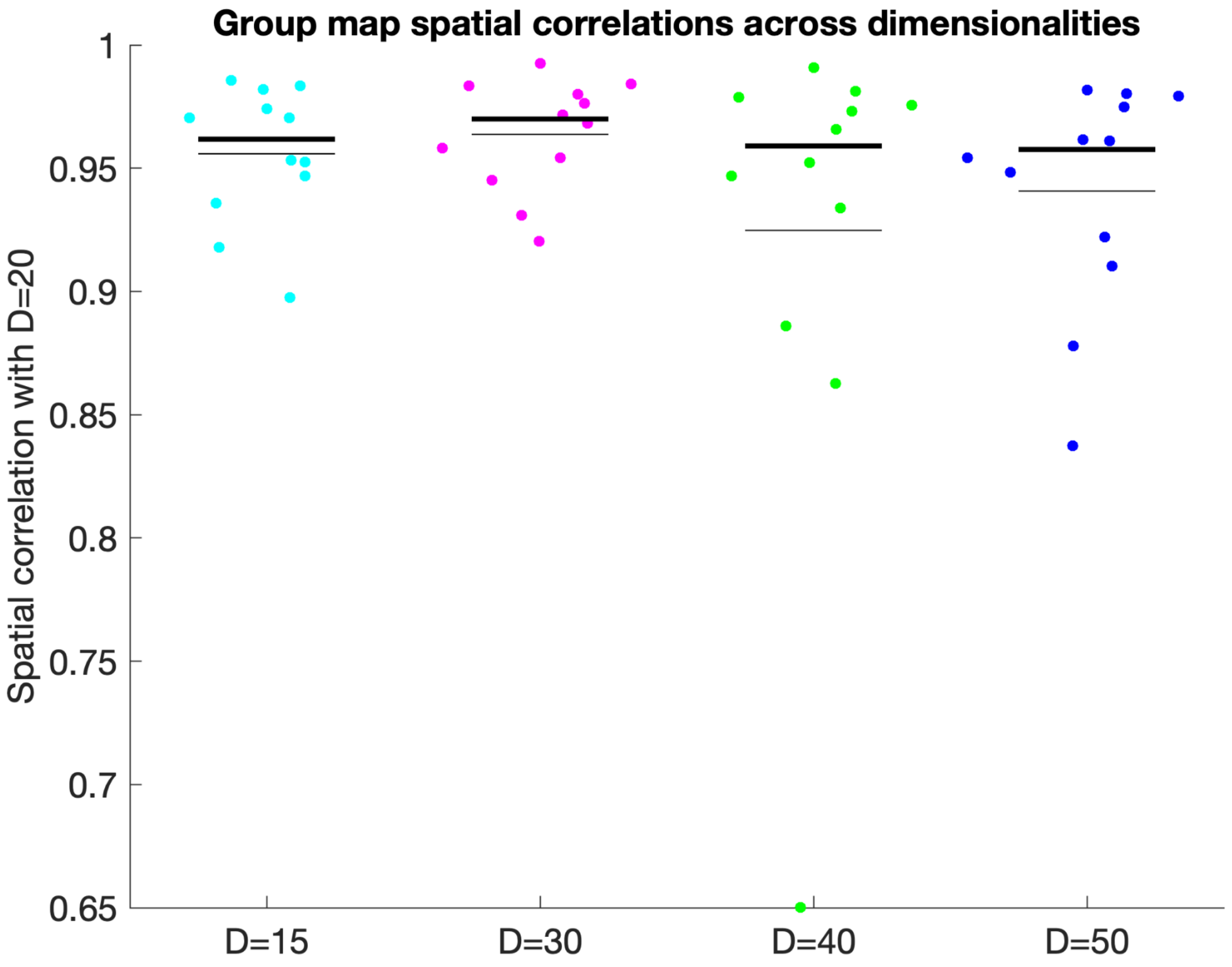

The resting state networks investigated in this article were consistently found across a range of PFM dimensionalities.

Figure S4: Missing modes

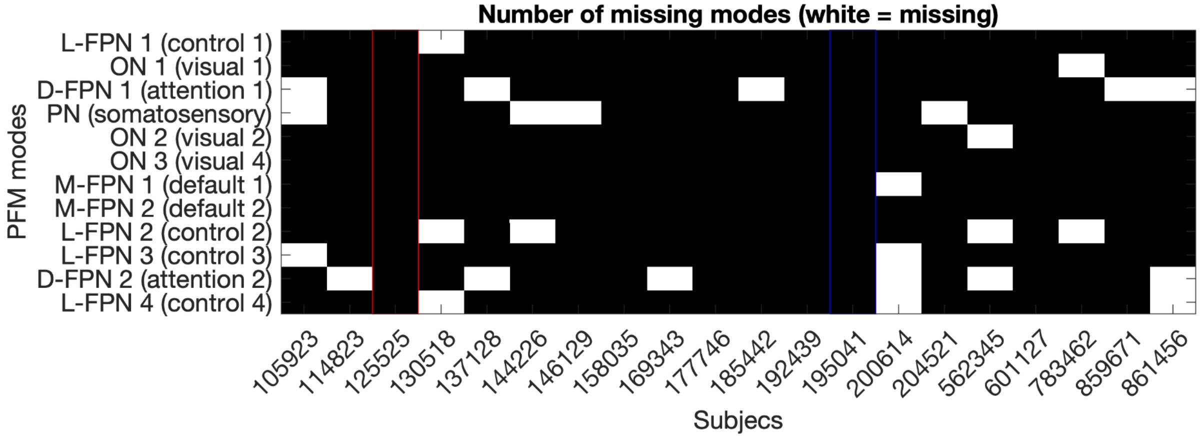

Missing (non-estimable) modes for each subject based on the individual subject PROFUMO runs are shown in white. Red and blue columns indicate the examples participants used throughout this paper.

Figure S5: Subject maps

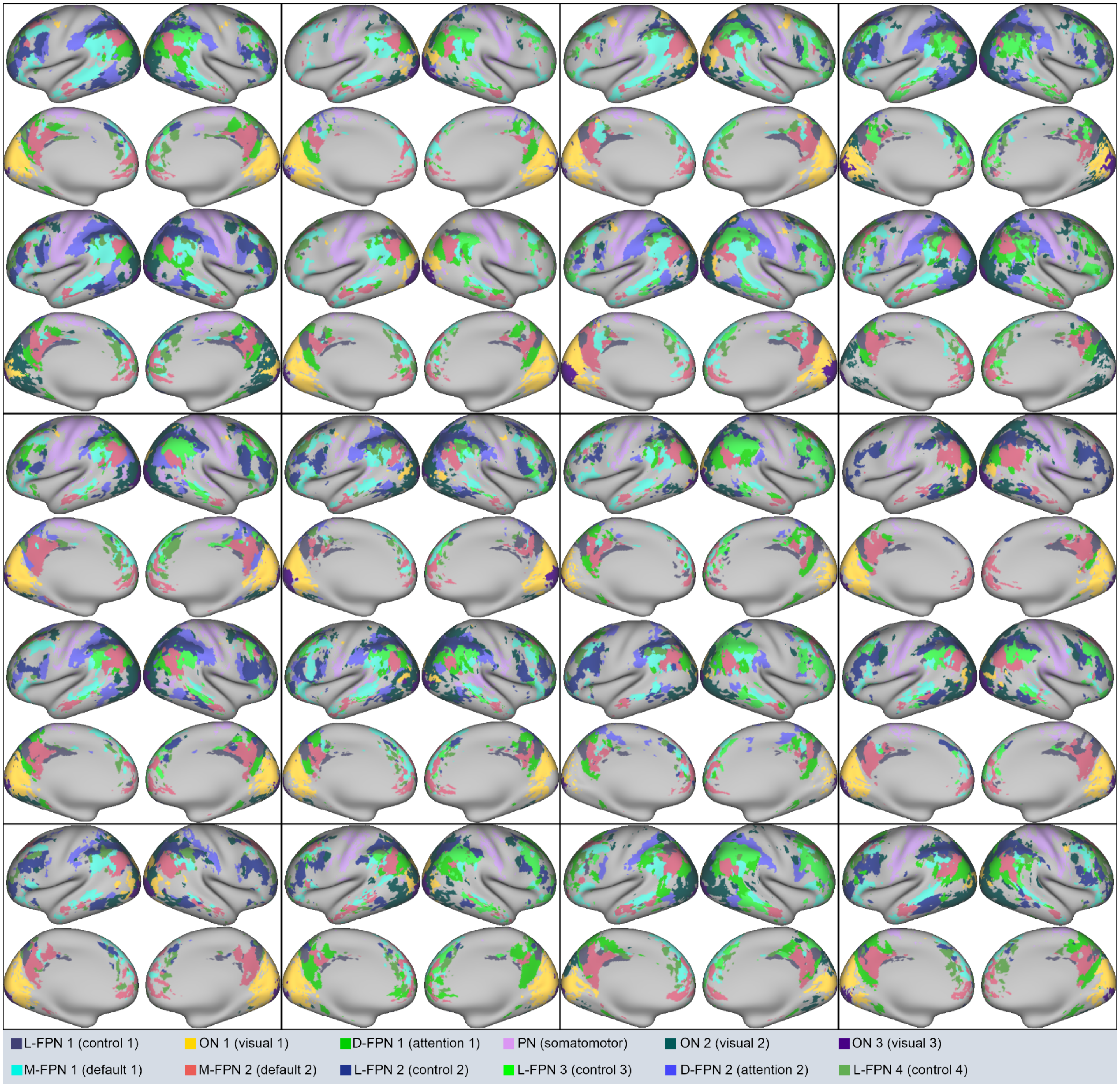

Subject maps from individual subject PROFUMO runs. Vertices were allocated to the strongest network (i.e., overlap is not shown; see Fig. S5 for overlap maps). Vertices with a weight lower than one for each of the twelve modes were unassigned to focus on robust networks. Twin pairs are grouped vertically within each rectangle for the top rows and non-twin individual participants are shown on the bottom row. For naming purposes, modes were spatially mapped onto the Yeo-7 parcellation (Yeo et al., 2011) and we followed the naming convention from (Uddin et al., 2019). FPN = frontoparietal network; L = lateral; D = dorsal; M = medial; ON = occipital network; PN = pericentral network.

Figure S6: Unthresholded version of spatial similarity figure

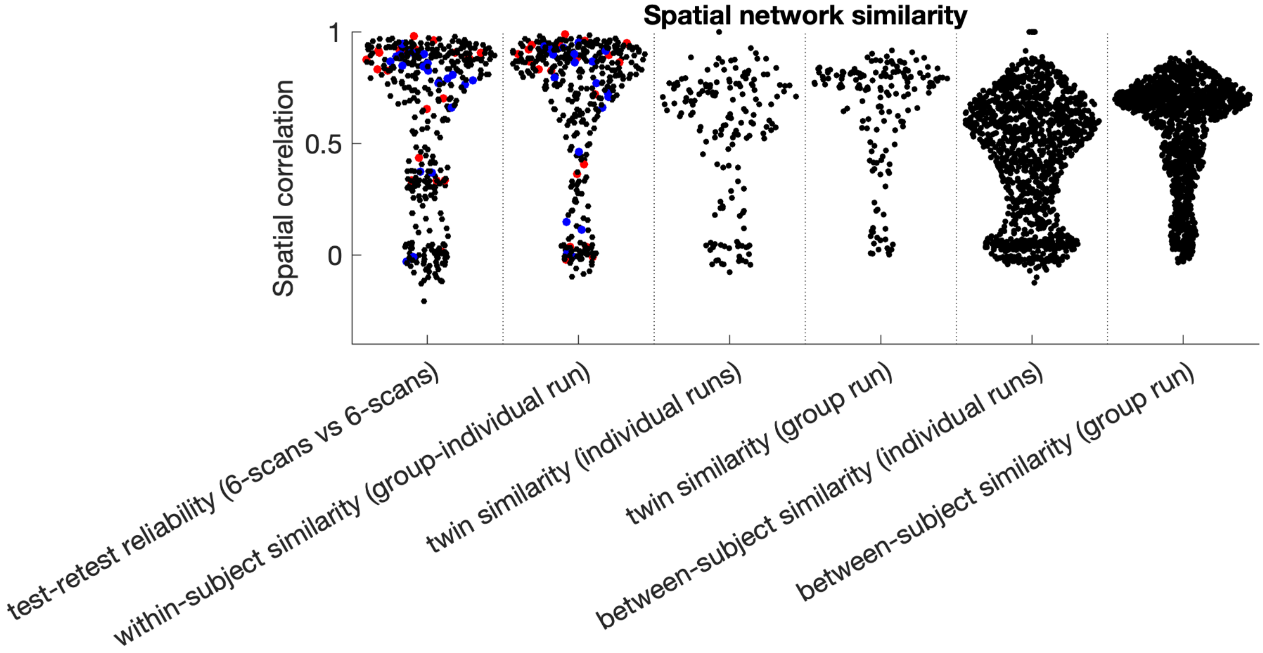

Version of figure 5 in the main manuscript without removing any PROFUMO modes (either as missing modes at the individual participant level or as non-replicable modes at the group level). As expected, the tails of the distributions are heavier than observed in Fig. 5, but the column-wise differences remain evident.

Figure S7: Stability of temporal connectivity matrices

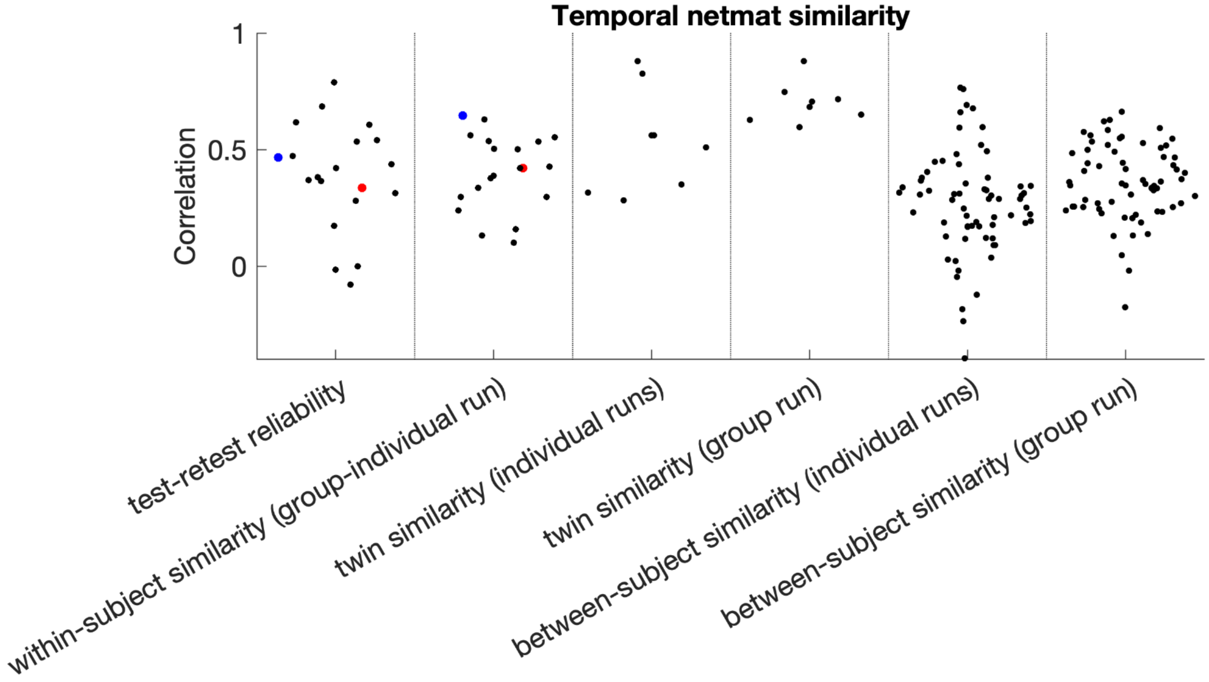

Stability of temporal connectivity matrix (also known as network matrix or 'netmat'). Red and blue dots indicate results from the examples participants used throughout this paper.

Figure S8: Stability of spatial overlap matrices

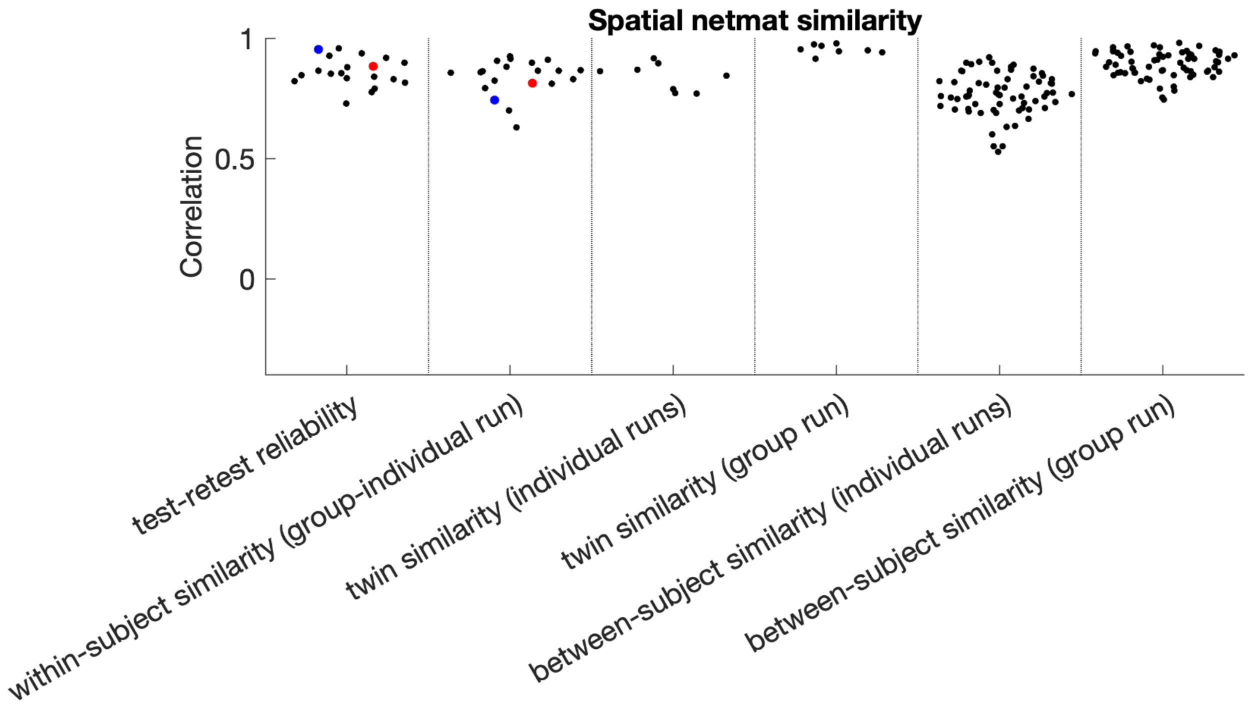

Stability of spatial overlap matrix. Red and blue dots indicate results from the examples participants used throughout this paper.

Figure S9: Spatial overlap maps for all participants

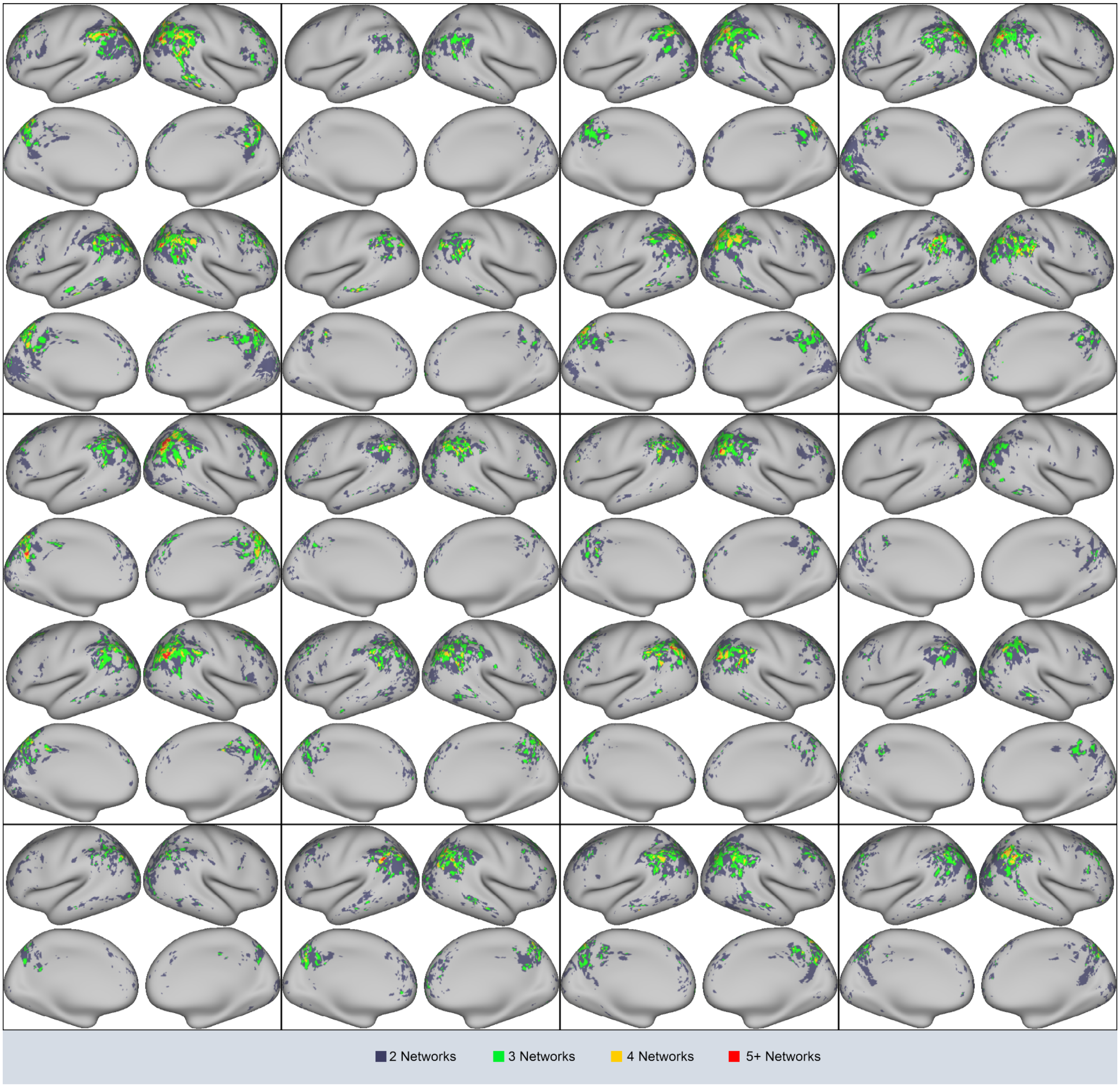

Overlap maps for all participants. Twin pairs are grouped vertically within each rectangle for the top rows and non-twin individual participants are shown on the bottom row.

Figure S10: Timeseries similarity as a function of thresholds

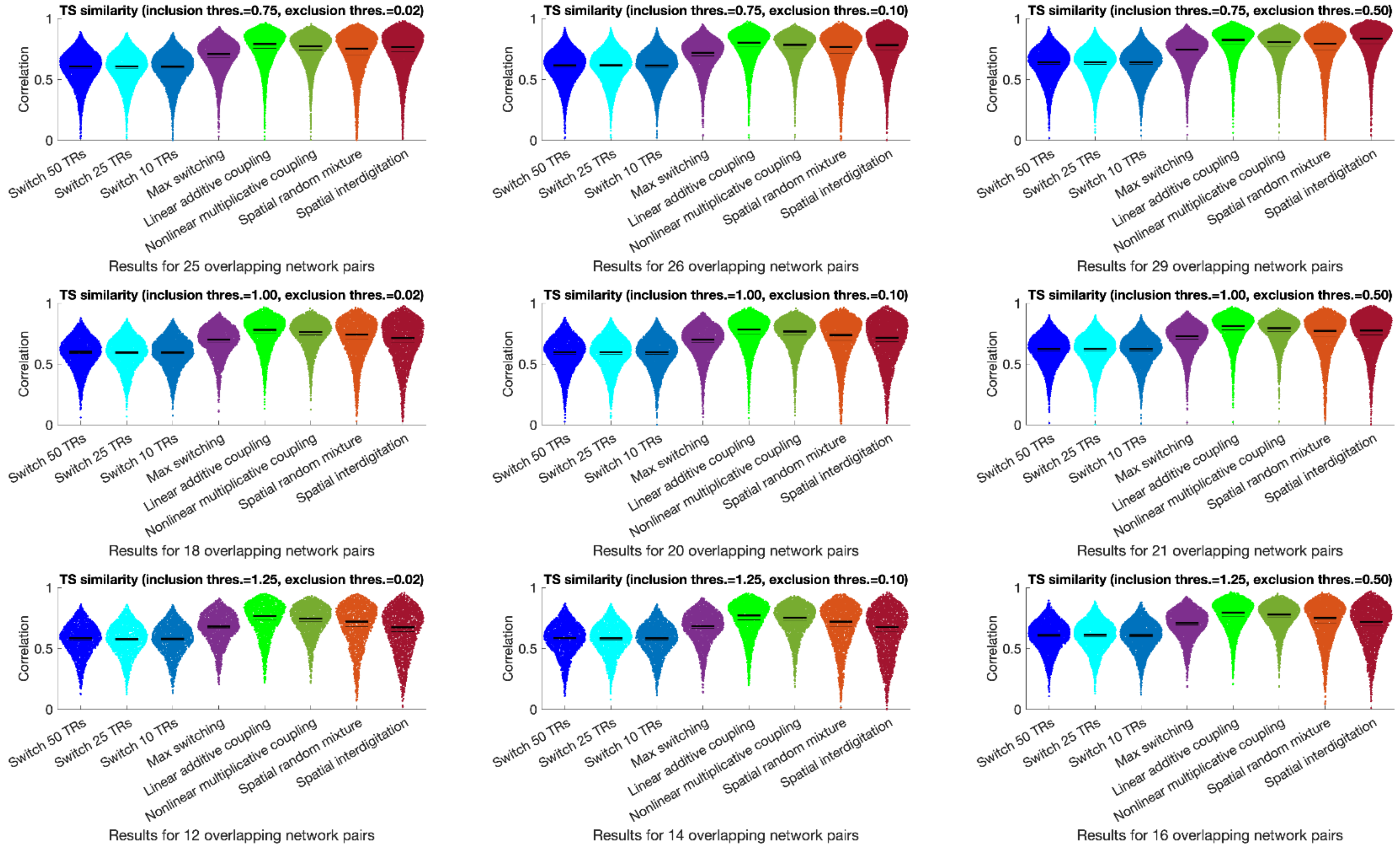

Repetitions of Figure 8 from the main manuscript across a range of thresholds for network inclusion and exclusion. Three thresholds were adopted to determine network extent [0.75 1 1.25] shown in rows, and three thresholds were adopted to limit contributions from other networks [0.02 0.1 0.5] shown in columns. The linear additive model shown in bright green consistently achieves the highest or equal highest median timeseries correlation regardless of thresholds.

Figure S11: Timeseries similarity displayed separately for each network pair

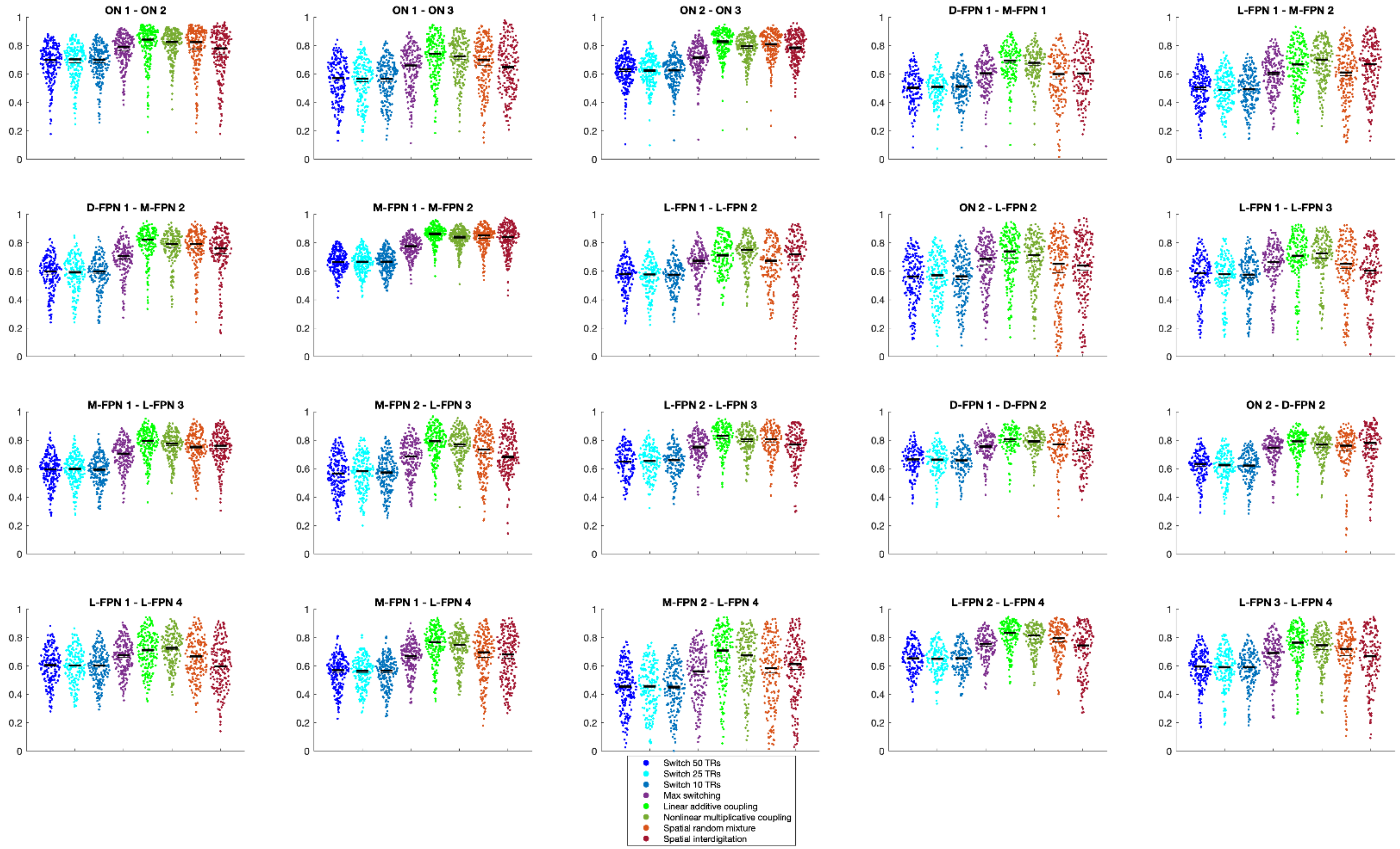

Version of Figure 8 from the main manuscript with results shown separately for each of the 20 overlapping networkk pairs. Y-axis shows correlations between the true overlap timeseries and different versions of the simulated overlap timeseries. Thin black lines indicate the mean and thick black lines indicate the median. Highest similarity was consistently observed for the linear additive coupling simulated overlap timeseries in bright green.

Figure S12: Frequency characteristics of semi-simulated timeseries

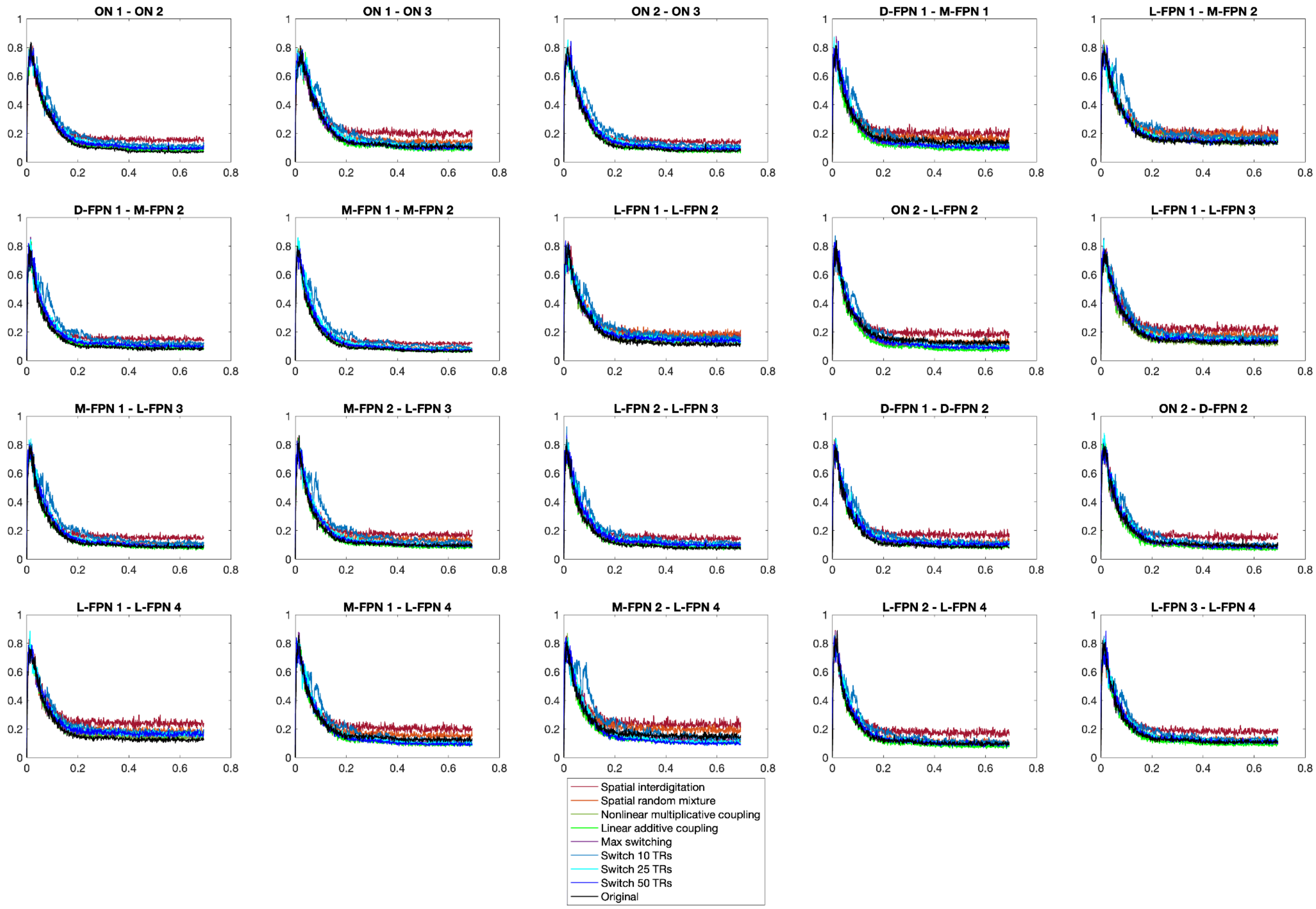

References

Uddin, L.Q., Yeo, B.T.T., Spreng, R.N., 2019. Towards a Universal Taxonomy of Macro-scale Functional Human Brain Networks. Brain Topogr. https://doi.org/10.1007/s10548-019-00744-6

Yeo, B.T.T., Krienen, F.M., Sepulcre, J., Sabuncu, M.R., Lashkari, D., Hollinshead, M., Roffman, J.L., Smoller, J.W., Zöllei, L., Polimeni, J.R., Fischl, B., Liu, H., Buckner, R.L., 2011. The organization of the human cerebral cortex estimated by intrinsic functional connectivity. J. Neurophysiol. 106, 1125–1165.
